# Supplementary material for: Uralenol, Glycyrol, and Abyssinone II as potent inhibitors of fibroblast growth factor receptor 2 from anti-cancer plants: A deep learning and molecular dynamics approach
Source: PLoS One. 2026 Jan 30;21(1):e0341498. doi: 10.1371/journal.pone.0341498 (PMC12857956; doi:10.1371/journal.pone.0341498)
Supplement: S1 Table — (DOCX) [file pone.0341498.s001.docx]

**S1 Table.** List of anti-cancer properties of medicinal plants with their 1350

Phytochemicals

| **Serial no.** | **Plants** | **Phytochemicals** | | | | | | | **Pubchem CID** | | | | | **Reference** |
| --- | --- | --- | --- | --- | --- | --- | --- | --- | --- | --- | --- | --- | --- | --- |
| 1 | *Peganum harmala* L. | \| p-Cymene \| 7463 \| \| --- \| --- \| \| Limonene \| 22311 \| \| 2-Acetyl-Thiazole \| 520108 \| \| Santolinaalcohol \| 241064929 \| \| n-Octanol \| 957 \| \| Linalool \| 6549 \| \| trans-Thujone \| 91456 \| \| 3-Decanone \| 13576 \| \| Camphor \| 2537 \| \| Benzeneacetonitrile \| 8794 \| \| Isoborneol \| 6321405 \| \| Terpinen-4-ol \| 11230 \| \| Naphthalene \| 931 \| \| Terpineol \| 17100 \| \| 1-Dodecene \| 8183 \| \| Methylchavicol \| 8815 \| \| Isoquinoline \| 8405 \| \| Carvone \| 7439 \| \| Pulegone \| 442495 \| \| Cuminaldehyde \| 326 \| \| (E)-Anethol \| 637563 \| \| Thymol \| 6989 \| \| 4-Methoxyacetophenone \| 7476 \| \| Eugenol \| 3314 \| \| Ylangene \| 20055075 \| \| Cubebene \| 518814 \| \| n-Undecanol \| 8184 \| \| iso-Italicene \| 10987385 \| \| Longipinene \| 12311396 \| \| Methyleugenol \| 7127 \| \| Ionol \| 31404 \| \| (Z)-Caryophyllene \| 6429301 \| \| (E)--Ionone \| 638014 \| \| Aromadendrene \| 11095734 \| \| 9-epi-(E)-Caryophyllene \| 6429274 \| \| Acoradiene \| 90351 \| \| Gurjunene \| 15560275 \| \| Muurolene \| 12306047 \| \| (E)--Ionone \| 638014 \| \| (E)-Methylisoeugenol \| 637776 \| \| Zingiberene \| 92776 \| \| Amorphene \| 12306046 \| \| Curcumene \| 92139 \| \| 7-epi--Selinene \| 91753195 \| \| Eugenolacetate \| 3606373 \| \| Bulnesene \| 16679399 \| \| (Z)-Nerolidol \| 8888 \| \| Calacorene \| 528708 \| \| GermacreneB \| 6370843 \| \| Spathulenol \| 92231 \| \| Caryophylleneoxide \| 1742210 \| \| Ledol \| 92812 \| \| Oplopenone \| 6429350 \| \| Cubenol \| 11770062 \| \| epi--Cadinol \| 160799 \| \| n-Tetradecanol \| 8209 \| \| Longifolol \| 12311097 \| \| Farnesol \| 445070 \| \| Lanceol \| 15560069 \| | | | | | | | | | | | | ^1^ |
|  |  | α-Pinene | | | | | 6654 | | | | | | | ^2^ |
|  |  | Styrene | | | | | 7501 | | | | | | |  |
|  |  | trans-Verbenole | | | | | 643101 | | | | | | |  |
|  |  | Sabinene | | | | | 18818 | | | | | | |  |
| 2 | *Curcuma longa* L. | \| alphaPinene \| 6654 \| \| --- \| --- \| \| Myrcene \| 31253 \| \| alphaTerpinene \| 7462 \| \| p-Cymene \| 7463 \| \| Beta-Phellandrene \| 11142 \| \| 1,8-Cineole \| 2758 \| \| Terpinolene \| 11463 \| \| alphaTerpineol \| 17100 \| \| Beta-Caryophyllene \| 5281515 \| \| (E)-Beta-Farnesene \| 5281517 \| \| ar-Curcumene \| 3083834 \| \| Beta-Bisabolene \| 10104370 \| \| Beta-Sesquiphellandrene \| 12315492 \| \| ar-Turmerol \| 5315469 \| \| Curzerenone \| 3081930 \| \| Beta-Eudesmol \| 91457 \| \| ar-Turmerone \| 160512 \| \| Germacrone \| 6436348 \| \| Beta-Turmerone \| 196216 \| \| (6R,7R)-Bisabolone \| 11321983 \| | | | | | | | | | | | | ^3^ |
|  |  | \| Curcumin \| 969516 \| \| --- \| --- \| \| Ar-turmerone \| 160512 \| \| Methylcurcumin \| 71717791 \| \| Demethoxy curcumin \| 5469424 \| \| Bisdemethoxy curcumin \| 5315472 \| \| Sodium curcuminate \| 6445487 \| | | | | | | | | | | | | ^4^ |
|  |  | Curlone | | | 196216 | | | | | | | | | ^5^ |
|  |  | Zingiberene | | | 92776 | | | | | | | | |  |
|  |  | Alpha-phellandrene | | | 7460 | | | | | | | | |  |
| 3 | *Allium wallichii* Kunth | \| Methane thiobis \| 1068 \| \| --- \| --- \| \| Propanal, 2-methyl \| 527 \| \| Ethanol \| 702 \| \| Ethylene oxide \| 6354 \| \| Butane, 2-bromo \| 6554 \| \| Pentanal \| 8063 \| \| Furane, 2-ethyl \| 18554 \| \| Disulfide, dimethyl \| 12232 \| \| Thiophene, 2,4-dimethyl \| 34296 \| \| Disulfide, methyl propyl \| 16592 \| \| 1,3-Oxathiane, 2,2-dimethyl \| 534530 \| \| Disulfide, 2-propenyl-propyl \| 16591 \| \| 4-Pentenal \| 16418 \| \| Propane \| 6334 \| \| Hexanal \| 6184 \| \| Oxetanone, 4,4-dimethyl \| 136214 \| \| 1-Propene, 3,3,3-trifluoro-2-methyl \| 136214 \| \| 2-Furan carboxaldehyde \| 7362 \| \| 2,5-Cyclohexadien-1-one, 4-ethyl-3,4-dimethyl \| 572122 \| \| 2-Hexanal \| 11583 \| \| Thiophene, 2,4-dimethyl \| 34296 \| \| 3,4-Pentadienal, 2,2-dimethyl \| 77678 \| \| Thiophene, 2,4-dimethyl \| 34296 \| \| 1-Propene, 3,3'[thiobis] \| 11617 \| \| Disulfide, methyl propyl \| 16592 \| \| Ethene, 1,2-bis (methylthio) \| 5370886 \| \| Disulfide, methyl 1-propenyl \| 5366552 \| \| Benzaldehyde \| 240 \| \| Trisulfide dimethyl \| 19310 \| \| Cyclohexene, 4-methylene-1-(1-methylethyl) \| 66841 \| \| Furan, 2,3-dihydro-4-methyl \| 36744 \| \| Furan, 2-pentyl \| 19602 \| \| Beta-lphaMyrcene \|  \| \| Benzene, 1,4-dichloro \| 4685 \| \| Octane, 2,7-dimethyl \| 140597 \| \| Benzene, 1-methyl-4-(1-methyl ethyl) \| 527268 \| \| Cyclohexanone, 2,2,6-trimethyl \| 17000 \| \| Limonene \| 22311 \| \| 4-Octyne, 2-methyl \| 548928 \| \| Cyclopentasiloxane, decamethyl \| 10913 \| \| Trisulfide, dipropyl \| 22383 \| \| Thiophene, 2-methoxy-5-methyl \| 141615 \| \| Isothiazole, 4,5-dimethyl \| 520305 \| \| Thieno[2,3-b][1]benzothiophene \| 605317 \| \| 2H-Thiopyran-3(6H)-one \| 534113 \| | | | | | | | | | | | | ^6^ |
|  |  | Dimethyl trisulfide | | | | 19416 | | | | | | | | ^7^ |
|  |  | Diosgenin | | | | 99474 | | | | | | | |  |
|  |  | Tigogenin | | | | 99516 | | | | | | | |  |
|  |  | 2,4-Dimethylthiophene | | | | 12053 | | | | | | | |  |
| 4 | *Artemisia annua* L. | \| Ethyl2-methylbutanoate \| 24020 \| \| --- \| --- \| \| Artemisiatriene \| 5320377 \| \| Camphene \| 6616 \| \| Sabinene \| 18818 \| \| 1,8-Cineole \| 2758 \| \| cis-Sabinenehydrate \| 62367 \| \| trans-Sabinenehydrate \| 6431628 \| \| trans-Pinocarveol \| 88302 \| \| Camphor \| 2537 \| \| Pinocarvone \| 121719 \| \| Borneol \| 64685 \| \| Terpinen-4-ol \| 11230 \| \| p-Cymen-8-ol \| 14529 \| \| Alpha-Terpineol \| 17100 \| \| Myrtenal \| 61130 \| \| Myrtenol \| 10582 \| \| 3-Tetradecene \| 5352802 \| \| p-Cymen-7-ol \| 325 \| \| Piperitenone \| 381152 \| \| Eugenol \| 3314 \| \| Alpha-Copaene \| 19725 \| \| Beta-Elemene \| 6918391 \| \| Beta-Caryophyllene \| 5281515 \| \| Alpha-Humulene \| 5281520 \| \| (E)-Beta-Farnesene \| 5281517 \| \| τ-Selinene \| 12308845 \| \| Germacrene-D \| 91723653 \| \| Beta-Selinene \| 442393 \| \| Viridiflorene \| 10910653 \| \| Isobornyl-3-methylbutanoate \| 36690486 \| \| Caryophylleneoxide \| 1742210 \| \| 1-epi-Cubenol \| 519857 \| \| Selin-11-en-4-Alpha-ol \| 15560330 \| \| Alpha-Bisabolol \| 1549992 \| \| Phytol \| 5280435 \| | | | | | | | | | | | | ^8^ |
|  |  | Isopinocamphone | | | | 84532 | | | | | | | | ^9^ |
|  |  | Neryl acetate | | | | 1549025 | | | | | | | |  |
|  |  | Germacrene-D | | | | 91723653 | | | | | | | |  |
| 5 | *Camellia sinensis* (L.) Kuntze | \| 1-Octen-3-ol \| 18827 \| \| --- \| --- \| \| Myrcene \| 31253 \| \| Limonene \| 22311 \| \| Ocimene \| 5281553 \| \| Linalool \| 6549 \| \| Nonanal \| 31289 \| \| Phenylethyl alcohol \| 6054 \| \| Linalool oxide (pyranoid) \| 26396 \| \| Naphthalene \| 931 \| \| (Z)-3-Hexenyl butyrate \| 5352438 \| \| Methyl salicylate \| 4133 \| \| Safranal \| 61041 \| \| Dodecane \| 8182 \| \| Decanal \| 8175 \| \| Geraniol \| 637566 \| \| Hexanoic acid, anhydride \| 74918 \| \| Indole \| 798 \| \| Tridecane \| 12388 \| \| 2-Methyl naphthalene \| 7055 \| \| Methyl anthranilate \| 8635 \| \| Alpha-Cubebene \| 442359 \| \| Alpha-Ionene \| 68057 \| \| Copaene \| 12303902 \| \| (Z)-3-hexenyl hexanoate \| 5352543 \| \| Hexyl hexanoate \| 22873 \| \| (E)-2-Hexenyl hexanoate \| 5352973 \| \| Jasmone \| 1549018 \| \| Tetradecane \| 12389 \| \| Longifolene \| 289151 \| \| Cedrene \| 521207 \| \| Beta-Caryophyllene \| 5281515 \| \| Alpha-Ionone \| 5282108 \| \| Geranyl acetone \| 1549778 \| \| Beta-Farnesene \| 5281517 \| \| γ-Muurolene \| 12313020 \| \| Alpha-Curcumene \| 92139 \| \| Beta-Ionone \| 638014 \| \| Pentadecane \| 12391 \| \| Alpha-Farnesene \| 5281516 \| \| γ-Cadinene \| 6432404 \| \| δ-Cadinene \| 441005 \| \| Nerolidol \| 5284507 \| \| 1-Hexadecene \| 12395 \| \| Hexadecane \| 11006 \| \| Heptadecane \| 12398 \| \| Octadecane \| 11635 \| \| Caffeine \| 2519 \| | | | | | | | | | | | | ^10^ |
|  |  | 2-Pentadecanone, 6, 10, 14-trimethyl | | | 10408 | | | | | | | | | ^11^ |
|  |  | Hexadecanoic acid, methyl ester | | | 8181 | | | | | | | | |  |
|  |  | 9,12-Octadecadienoic acid, methyl ester | | | 5284421 | | | | | | | | |  |
|  |  | 9, 12, 15-Octadecatrienoic acid | | | 5362857 | | | | | | | | |  |
|  |  | Heneicosanoic acid, methyl ester | | | 22434 | | | | | | | | |  |
| 6 | *Paeonia suffruticosa* Andr. | \| Tetradecanoic acid, methyl ester \| 31284 \| \| --- \| --- \| \| cis‐5‐Dodecenoic acid, methyl ester \| 14524604 \| \| Pentadecanoic acid, methyl ester \| 23518 \| \| Hexadecanoic acid, methyl ester \| 8181 \| \| (9Z, 12Z)‐Octadecadienoic acid, methyl ester \| 5284421 \| \| (9Z, 12Z, 15Z)‐Octadecatrienoic acid, methyl ester \| 5319706 \| \| Resveratrol \| 445154 \| \| Paeoniflorin \| 442534 \| \| Luteolin \| 5280445 \| \| Apigenin \| 5280443 \| \| Kaempferol \| 5280863 \| \| Oleanic acid \| 10494 \| \| Hederagenin \| 73299 \| \| Caffeic acid \| 689043 \| \| Kojic acid \| 3840 \| \| Acarbose \| 41774 \| \| Huperzine A \| 854026 \| | | | | | | | | | | | | ^12^ |
|  |  | Palmitic acid | | | 985 | | | | | | | | | ^13^ |
|  |  | Palmitic acid | | | 985 | | | | | | | | |  |
|  |  | Stearic acid | | | 5281 | | | | | | | | |  |
|  |  | LA (Linoleic acid) | | | 5280450 | | | | | | | | |  |
|  |  | ALA (α-Linolenic acid) | | | 5280934 | | | | | | | | |  |
| 7 | *Ocimum sanctum* L. | \| Vicenin 2 \| 442664 \| \| --- \| --- \| \| Luteolin-7-O-glucuronide \| 13607752 \| \| Isoorientin \| 114776 \| \| Orientin \| 5281675 \| \| Apigenin-7-O-glucuronide \| 5319484 \| \| Vitexin \| 5280441 \| \| Isovitexin \| 162350 \| \| Rosmarinic acid \| 5281792 \| \| Chlorogenic acid \| 1794427 \| \| Aesculin \| 5281417 \| \| Quercetin \| 5280343 \| \| Luteolin \| 5280445 \| \| Apigenin \| 5280443 \| \| Caffeic acid \| 689043 \| \| Cirsimaritin \| 188323 \| | | | | | | | | | | | | ^14^ |
|  |  | \| Pentanal \| 8063 \| \| --- \| --- \| \| Sotolone \| 62835 \| \| Caryophyllene \| 5281515 \| \| Isoeugenol \| 853433 \| \| Eugenol \| 3314 \| | | | | | | | | | | | | ^15^ |
|  |  | Eugenol | | | | | | 3314 | | | | | | ^16^ |
|  |  | alpha-Farnesene | | | | | | 5281516 | | | | | |  |
|  |  | Cyclohexane,1,2,4- triethenyl | | | | | | 96529 | | | | | |  |
| 8 | *Punica granatum* L. | \| Punicic acid \| 5281126 \| \| --- \| --- \| \| myricetin \| 5281672 \| \| kaempferol \| 5280863 \| \| luteolin \| 5280445 \| \| brevifolin \| 66654 \| \| isolariciresinol \| 160521 \| \| 𝛼-conidendrin \| 457194 \| \| pelletierine \| 92987 \| \| ursolic acid \| 64945 \| \| phloretin \| 4788 \| \| coumestrol \| 5281707 \| \| asiatic acid \| 119034 \| \| isopelletierine \| 92987 \| \| matairesinol \| 119205 \| \| hovetrichoside-C \| 42607791 \| \| betulinic acid \| 64971 \| \| pseudopelletierine \| 11096 \| \| methionine \| 6137 \| \| vanillic acid \| 8468 \| \| corilagin \| 73568 \| \| chlorogenic acid \| 1794427 \| \| neochlorogenic acid \| 5280633 \| \| hippomanin-A \| 323958 \| \| sinapic acid \| 637775 \| \| castalagin \| 168165 \| \| isocorilagin \| 10077799 \| \| maslinic acid \| 73659 \| \| oenothein-B \| 16129800 \| | | | | | | | | | | | | ^17^ |
|  |  | Furfural | | | | | 7362 | | | | | | | ^18^ |
|  |  | 2H-Pyran-2-one | | | | | 643938 | | | | | | |  |
|  |  | Furan | | | | | 2977 | | | | | | |  |
|  |  | Furyl hydroxymethyl ketone | | | | | 8131 | | | | | | |  |
|  |  | Methyl 2-furoate | | | | | 12128 | | | | | | |  |
|  |  | 2(1H)-Pyridinone | | | | | 5117 | | | | | | |  |
|  |  | 1-Propanone, 1-(2-furanyl)- | | | | | 61168 | | | | | | |  |
|  |  | 3-Furancarboxylic acid, methyl ester | | | | | 76775 | | | | | | |  |
|  |  | 4H-Pyran-4-one, 2,3-dihydro-3,5-dihydroxy-6-methyl- | | | | | 102553 | | | | | | |  |
|  |  | 5-Hydroxymethylfurfural | | | | | 237332 | | | | | | |  |
|  |  | Benzenemethanol, 3-fluoro- | | | | | 474 | | | | | | |  |
|  |  | 3,5-Dimethyl-3-heptene | | | | | 122295 | | | | | | |  |
|  |  | 4-Mercaptophenol | | | | | 962 | | | | | | |  |
|  |  | 4-Fluorobenzyl alcohol | | | | | 10170 | | | | | | |  |
|  |  | Thiophene, 2-propyl- | | | | | 15217 | | | | | | |  |
|  |  | Nonanoic acid, 2,4,6-trimethyl-, methyl ester, (2R,4S,6R)-(-)- | | | | | 579737 | | | | | | |  |
|  |  | Ethyl 13-methyl-tetradecanoate | | | | | 549646 | | | | | | |  |
|  |  | 9-Octadecenoic acid (Z)-, methyl ester (Methyl oleate) | | | | | 8180 | | | | | | |  |
|  |  | Hexadecanoic acid, ethyl ester (Ethyl palmitate) | 8159 | | | | | | | | | | |  |
|  |  | l-Docosanethiol (1-Docosanethiol) | 16733 | | | | | | | | | | |  |
|  |  | Heptadecanoic acid, ethyl ester | 33261 | | | | | | | | | | |  |
|  |  | Ethyl oleate | 5365538 | | | | | | | | | | |  |
|  |  | Octadecanoic acid, ethyl ester (Ethyl stearate) | 8121 | | | | | | | | | | |  |
|  |  | Iridomyrmecin | 10237 | | | | | | | | | | |  |
|  |  | Oleyl alcohol, heptafluorobutyrate | 578665 | | | | | | | | | | |  |
|  |  | 1,12-Bis(2-nitrophenoxy)dodecane | 454174 | | | | | | | | | | |  |
|  |  | Acetic acid | 176 | | | | | | | | | | |  |
|  |  | Octadecane | 11525 | | | | | | | | | | |  |
|  |  | 1-ethyl-2-hydroxymethyl imidazole | 157291 | | | | | | | | | | |  |
|  |  | Bamipine | 2293 | | | | | | | | | | |  |
|  |  | Oleic Acid | 445639 | | | | | | | | | | |  |
|  |  | Linoleic acid ethyl ester | 5282583 | | | | | | | | | | |  |
|  |  | 2-Hexyne | 138339 | | | | | | | | | | |  |
|  |  | 6-Methyl-3,5-Heptadien-2-One | 5366472 | | | | | | | | | | |  |
|  |  | 1,4-Hexadiene, 4-methyl- | 5354966 | | | | | | | | | | |  |
|  |  | 9,12,15-Octadecatrienoic acid, ethyl ester, (Z,Z,Z)- | 5282572 | | | | | | | | | | |  |
|  |  | 2-Cyclohexen-1-one | 13627 | | | | | | | | | | |  |
| 9 | *Ginkgo biloba* L. | \| Tetradecanoic acid \| 11005 \| \| --- \| --- \| \| Hexadecanoic acid \| 985 \| \| 9,12-Octadecadienoic acid \| 3931 \| \| 9,12,15-Octadecatrienoic acid \| 5280934 \| \| Octadecane \| 11635 \| \| Beta-Sitosterol \| 222284 \| | | | | | | | | | | | | ^19^ |
|  |  | Phytadiene (Neophytadiene) | | | | | | | | 5352800 | | | | ^20^ |
|  |  | Heptadecanoic acid | | | | | | | | 10465 | | | |  |
|  |  | Eicosanoic acid | | | | | | | | 10467 | | | |  |
|  |  | Docosanoic acid | | | | | | | | 10465 | | | |  |
|  |  | Tetracosanoic acid | | | | | | | | 11197 | | | |  |
|  |  | Hexacosanoic acid | | | | | | | | 10469 | | | |  |
|  |  | Octacosanoic acid | | | | | | | | 10471 | | | |  |
|  |  | 10-Nonacosanol | | | | | | | | 108097 | | | |  |
|  |  | Phytadiene (Neophytadiene) | | | | | | | | 5352800 | | | |  |
| 10 | *Ziziphus mauritiana* Lam. | \| Myricetin 3-O-galactoside \| 5491408 \| \| --- \| --- \| \| Quercetin 3-O-pentosylhexoside \|  \| \| Quercetin 3-O-robinobioside \| 10371536 \| \| Quercetin 3-O-glucoside \| 5280804 \| \| Quercetin 3-O-rhamnoside \| 5280459 \| \| Luteolin 7-O-6-malonyl glucoside \| 5281669 \| \| Epicatechin \| 72276 \| \| Quercetin \| 5280343 \| \| Quercetin 3-O-rutinoside \| 5280805 \| \| Quercetin 3-O-galactoside \| 5281643 \| \| Naringenin and naringenin glycoside \| 442428 \| | | | | | | | | | | | | ^21^ |
|  |  | \| Thymine \| 1135 \| \| --- \| --- \| \| Molinate \| 16653 \| \| Clindamycin \| 446598 \| \| Levetiracetam \| 5284583 \| \| Maltol \| 8369 \| \| Octanoic Acid, 2-Hexyl- \| 109035 \| \| 2-Propyl-octanoic acid \| 57567206 \| \| 5-Hydroxymethylfurfural \| 237332 \| \| Pentanoic acid, nonyl ester \| 568602 \| \| Malonic acid, ethyl \| 7761 \| \| D-Allose \| 439507 \| \| Beta-D-Glucopyranoside, methyl \| 445238 \| \| 3,4-Altrosan \| 548229 \| \| Nonanoic acid \| 8158 \| \| 1,5-Anhydroglucitol \| 64960 \| \| Tetradecanoic acid \| 11005 \| \| Dodecanoic acid \| 3893 \| \| Oleic acid \| 445639 \| \| Stigmasterol \| 5280794 \| | | | | | | | | | | | | ^22^ |
|  |  | Formic acid, Heptyl ester (Heptyl formate) | | 8144 | | | | | | | | | | ^23^ |
|  |  | Glucose | | 5793 | | | | | | | | | |  |
|  |  | Polygalitol (1,5-Anhydroglucitol) | | 82283 | | | | | | | | | |  |
|  |  | 9-Octadecanoic acid, (E)- (Elaidic acid) | | 643841 | | | | | | | | | |  |
|  |  | Erucic acid | | 10505 | | | | | | | | | |  |
|  |  | Formic acid, Heptyl ester (Heptyl formate) | | 8144 | | | | | | | | | |  |
|  |  | \| Cyclopentasiloxane, decamethyl \| 10913 \| \| --- \| --- \| \| 2-Ethyl-1-butanol, methyl ether \| 10866310 \| \| 1.1.3.3.5.5.7.7-Octamethyl-1r-7-(2methyl-propoxy) tetrasiloxane-1 \|  \| \| 2-Pyrrolidinecarboxylic acid-5-oxo, ethyl ester \| 98047 \| \| 1-Dodecanamine, N,N-dimethyl \| 15433 \| \| 5-keto-2, 2-dimethylheptanoic acid, ethyl ester \| 545903 \| \| Cyclodecasiloxane, eicosamethyl \| 519601 \| \| 9,12,15-Octadecatrienoic acid \| 5280934 \| \| Octadecenal \| 5283381 \| \| 9-Octadecenamide \| 1930 \| \| N-Acetyl-L-tryptophan ethyl ester \| 2724382 \| | | | | | | | | | | | | ^24^ |
|  |  | α-Hydroxyisobutyric acid | | | | | | | | 11671 | | | | ^25^ |
|  |  | D-Lactic acid | | | | | | | | 61503 | | | |  |
|  |  | Hexonic acid (Hexanoic acid) | | | | | | | | 604 | | | |  |
|  |  | Ethanedioic acid (Oxalic acid) | | | | | 971 | | | | | | |  |
|  |  | 2-Deoxyribose | | | | | 5460005 | | | | | | |  |
|  |  | 1-O-Pentadecylglycerol | | | | | 91733515 | | | | | | |  |
|  |  | DL-Malic acid | | | | | 525 | | | | | | |  |
|  |  | Pyroglutamic acid | | | | | 7405 | | | | | | |  |
| 12 | *Anisomeles indica* (L.) Kuntze | \| Alpha-Pinene \| 6654 \| \| --- \| --- \| \| Camphene \| 6616 \| \| Beta-Pinene \| 14896 \| \| o-Cymene \| 10703 \| \| Cymol \| 7463 \| \| Limonene \| 22311 \| \| Nerol \| 643820 \| \| Thujene \| 18818 \| \| Sabinene \| 18818 \| \| Decanal \| 8175 \| \| Iso-bornyl acetate \| 443131 \| \| Eugenol \| 3314 \| \| Decyl ester of acetic acid \| 8167 \| \| Farnesene \| 5281516 \| | | | | | | | | | | | | ^26^ |
|  |  | \| Anisole \| 7519 \| \| --- \| --- \| \| Benzaldehyde \| 240 \| \| Trimethyl benzaldehyde \| 10254 \| \| Jasmatone \| 114454 \| \| Pygmaein \| 46911433 \| \| Cyclopentadecanolide \| 235414 \| \| Farnesyl acetone \| 1711945 \| \| Nootkatone \| 1268142 \| \| Phytol acetate \| 6428538 \| \| Methyl communate \| 11141639 \| \| Heptacosane \| 11636 \| \| Tritriacontane \| 12411 \| | | | | | | | | | | | | ^27^ |
|  |  | \| Hexyl butanoate \| 17525 \| \| --- \| --- \| \| p-Cymene \| 7463 \| \| Alpha-Ionone \| 5282108 \| \| (Z)-3-Octen-1-ol \| 5364519 \| \| Vitispirane \| 6450832 \| \| Beta-Caryophyllene \| 5281515 \| \| (E)-2-Octen-1-ol \| 5318599 \| \| Alpha-Terpineol \| 17100 \| \| Dehydro-ar-ionene \| 121677 \| \| Methyl salicylate \| 4133 \| \| (Z)-Beta-Damascenone \| 12309006 \| \| Hexyl butanoate \| 17525 \| \| p-Cymene \| 7463 \| | | | | | | | | | | | | ^28^ |
|  |  | \| Pedalitin (7-methoxy-3,4,5,6-tetrahydroxyflavone) \| 33172 \| \| --- \| --- \| \| Apigenin \| 5280443 \| \| Ovatodiolide \| 38347030 \| \| Methylgallate \| 7428 \| \| 3,4-Dihydroxybenzoic acid \| 72 \| \| Apigenin 7-O-glucuronide \| 5319484 \| \| Cistanoside F \| 101688189 \| \| Betonyoside A \| 274940264 \| \| Campneoside II \| 10009317 \| \| Acteoside \| 5281800 \| \| Isoacteoside \| 6476333 \| | | | | | | | | | | | | ^29^ |
| 13 | *Ziziphus spina-christi* (L.) Desf. | \| Methyl tetradecanoate \| 31284 \| \| --- \| --- \| \| 9-Hexadecenoic acid, methyl ester, (Z)- \| 643801 \| \| Hexadecanoic acid, methyl ester \| 8181 \| \| Hexadecanoic acid, 14-methyl-, methyl ester \| 520159 \| \| Squalene \| 638072 \| | | | | | | | | | | | | ^30^ |
|  |  | \| 3-Dodecene, (Z)- \| 5364450 \| \| --- \| --- \| \| 1-Tetradecene \| 14260 \| \| Butyl hydroxytoluene \| 31404 \| \| 1-Hexadecanol \| 2682 \| \| Dichloroacetic acid, tetradecyl ester \| 522798 \| \| 2,6,10,15-Tetramethyl heptadecane \| 41209 \| \| 1-Hexadecanol \| 2682 \| \| 7,9-Di-tert-butyl-1-oxaspiro(4,5)deca-6,9-diene-2,8-dione \| 545303 \| \| Betulin \| 9548595 \| \| Lanceol, cis (2E)-2-(4,7-Dimethyl-3,4,4a,5,6,8a-hexahydro-1(2H)-naphthalenylidene)-1-propanol \| 5352901 \| \| Hexadecanoic acid, methyl ester (Palmitic methyl ester) \| 8181 \| \| 8,11-Octadecadienoic acid, methyl ester \| 5319737 \| \| Phytol (2-Hexadecen-1-ol, 3,7,11,15-tetramethyl-, (R-(R*,R*-(E)))) \| 5280435 \| \| Campesterol \| 173183 \| \| Stigmasterol \| 5280794 \| \| Ethyl iso-allocholate \| 6452096 \| \| 1-Butoxy-1-isobutoxy-butane \| 545190 \| \| 9-Octadecenoic acid (Z)-, oleic acid \| 445639 \| \| Phytol \| 5280435 \| | | | | | | | | | | | | ^31^ |
|  |  | Methyl tetradecanoate | | | | | | | | | 31284 | | | ^32^ |
|  |  | 9-Hexadecenoic acid, methyl ester, (Z)- | | | | | | | | | 14258 | | |  |
|  |  | Hexadecanoic acid, 14-methyl-, methyl ester | | | | | | | | | 520159 | | |  |
|  |  | Squalene | | | | | | | | | 638072 | | |  |
|  |  | 3-Dodecene, (Z)- | | | | | | | | | 5364450 | | |  |
|  |  | 1-Tetradecene | | | | | | | | | 14260 | | |  |
|  |  | Butyl hydroxytoluene | | | | | | | | | 31404 | | |  |
|  |  | Dichloroacetic acid, tetradecyl ester | | | | | | | | | 522798 | | |  |
|  |  | 2,6,10,15-Tetramethyl heptadecane | | | | | | | | | 132581818 | | |  |
|  |  | 7,9-Di-tert-butyl-1-oxaspiro(4,5)deca-6,9-diene-2,8-dione | | | | | | | | | 545303 | | |  |
| 14 | *Glycyrrhiza glabra* L. | \| Glycyrrhizin \| 14982 \| \| --- \| --- \| \| Glycyrrhetic acid \| 10114 \| \| Liquiritin \| 503737 \| \| Isoliquiritin \| 5318591 \| \| Glabridin \| 124052 \| | | | | | | | | | | | | ^33^ |
|  |  | \| Furfuraldehyde \| 7362 \| \| --- \| --- \| \| Licoarylcoumarin \| 10090416 \| \| Licoriphenone \| 21591149 \| \| 1-Methoxyficifolinol \| 480872 \| \| Shinpterocarpin \| 10336244 \| \| Alpha-Terpineol \| 17100 \| \| Prenyllicoflavone A \| 11349817 \| \| Liquiritigenin \| 114829 \| \| Isoliquiritigenin \| 638278 \| | | | | | | | | | | | | ^34^ |
|  |  | \| Methacrylonitrile \| 31368 \| \| --- \| --- \| \| Dehydroepiandrosterone \| 5881 \| \| Pyrazine, 1,4-dioxide \| 520103 \| \| 4-Aminopyridine \| 1727 \| \| 4-(2-Aminopropyl)phenol \| 3651 \| | | | | | | | | | | | | ^35^ |
|  |  | \| Octadecane \| 11635 \| \| --- \| --- \| \| Pentadecanoic acid \| 13849 \| \| 2,3,5,6-Tetramethylpyrazine \| 14296 \| \| 2-(4-Methylphenyl)propan-2-ol \| 14529 \| \| Undecane \| 14257 \| \| Benzyl Alcohol \| 244 \| \| 1-Butanol \| 263 \| \| Lauric acid \| 3893 \| \| Decanoic acid \| 2969 \| \| p-Cresol \| 2879 \| \| m-Cresol \| 342 \| \| Piperitenone \| 381152 \| \| Octanoic acid \| 379 \| \| Tetradecanal \| 31291 \| \| o-Cresol \| 335 \| \| Safrole \| 5144 \| \| 2-Pentylfuran \| 19602 \| \| p-Cymene \| 7463 \| \| Hexanal \| 6184 \| \| Thymol \| 6989 \| \| Methyleugenol \| 7127 \| \| Decanal \| 8175 \| \| Indole \| 798 \| \| Furfuryl alcohol \| 7361 \| \| Furfural \| 7362 \| \| Undecanoic acid \| 8180 \| \| Undecanal \| 8186 \| \| 1-Dodecanol \| 8193 \| \| 1-Hexanol \| 8103 \| \| Heptanal \| 8130 \| \| Heptanoic acid \| 8094 \| \| Dodecanal \| 8194 \| \| beta-Terpinene \| 66841 \| \| Ethyl acetate \| 8857 \| \| 1-Octanol \| 957 \| \| Heptadecane \| 12398 \| \| Nonadecane \| 12401 \| \| Docosane \| 12405 \| \| Heneicosane \| 12403 \| \| Pentadecane \| 12391 \| \| Tridecane \| 12388 \| \| Tridecanoic acid \| 12530 \| \| 1-Heptanol \| 8129 \| \| 2'-Hydroxyacetophenone \| 68490 \| \| 1-Undecanol \| 8184 \| \| Benzaldehyde \| 240 \| \| Dodecane \| 8182 \| \| Tetradecane \| 12389 \| \| 4-Carvomenthenol \| 11230 \| \| Terpinolene \| 11463 \| \| Pulegone \| 442495 \| \| (+)-delta-Cadinene \| 441005 \| \| Linalool \| 6549 \| \| Gamma-nonalactone \| 7710 \| \| Nonanoic acid \| 8158 \| \| alpha-Terpineol \| 17100 \| \| Ambrettolide \| 5365703 \| \| Menthone \| 26447 \| \| 3,5-Dimethylstyrene \| 21476 \| \| 2'-Methoxyacetophenone \| 77698 \| | | | | | | | | | | | | ^36^ |
| 15 | *Terminalia chebula* Retz. | \| 1,3,6-tri-O-galloyl-beta-D-glucose \| 452707 \| \| --- \| --- \| \| Bellericoside \| 273779104 \| \| Ascorbic acid \| 54670067 \| \| Pentagalloylglucose \| 65238 \| \| Docosanoic acid \| 8215 \| \| beta-Glucogallin \| 124021 \| \| Arachidic acid \| 10467 \| \| Arjunolic acid \| 73641 \| \| Arjugenin \| 12444386 \| \| arjunglucoside I \| 14658050 \| | | | | | | | | | | | | ^37^ |
|  |  | \| Punicalagin \| 16129719 \| \| --- \| --- \| \| Shikimic acid \| 8742 \| \| Palmitic acid \| 985 \| \| Ethyl gallate \| 13250 \| \| Gallic acid \| 370 \| \| terflavin A \| 16175788 \| \| beta-Sitosterol \| 222284 \| \| Daucosterol \| 5742590 \| \| Corosolic acid \| 6918774 \| \| Punicalin \| 92131301 \| \| Punicalagin \| 16129719 \| \| Terflavin C \| 274044461 \| \| Terflavin D \| 274040863 \| \| Maslinic acid \| 73659 \| \| Pyrogallol \| 1057 \| \| Vanillic acid \| 8468 \| \| Ferulic acid \| 445858 \| \| Caffeic acid \| 689043 \| | | | | | | | | | | | | ^38^ |
| 16 | *Mucuna pruriens* (L.) DC. | \| Bufotenine \| 10257 \| \| --- \| --- \| \| N,N-Dimethyl-5-methoxytryptamine \| 1832 \| \| Choline \| 305 \| \| N,N-Dimethyltryptamine \| 6089 \| \| 9H-Pyrido[3,4-B]indole \| 64961 \| \| Bufotenine \| 10257 \| \| 6-methoxy-1-methyl-9H-pyrido[3,4-b]indole \| 5376026 \| \| alpha-Amyrenyl acetate \| 92842 \| \| Acacetin \| 5280442 \| \| Luteolin \| 5280445 \| \| Ursolic acid \| 64945 \| \| Betulinic acid \| 64971 \| \| Stigmasterol \| 5280794 \| \| Nicotine \| 89594 \| \| (9Z)-(12S,13R)-12,13-Epoxyoctadecenoic acid \| 6449780 \| \| Levodopa \| 6047 \| | | | | | | | | | | | | ^39^ |
|  |  | \| Myristic acid \| 11005 \| \| --- \| --- \| \| Stearic acid \| 5281 \| \| Serotonin \| 5202 \| \| Arachidic acid \| 10467 \| \| Oleic acid \| 445639 \| \| Gallic acid \| 370 \| \| Linoleic acid \| 5280450 \| | | | | | | | | | | | | ^40^ |
|  |  | \| Propane,1,1-diethoxy-2-methyl \| 519415 \| \| --- \| --- \| \| 4-Heptanol,3-methyl- \| 102700 \| \| 1,2,3-Butanetriol \| 20497 \| \| Hexanoic acid \| 8892 \| \| Propane,1,1,3-triethoxy- \| 24624 \| \| 4-Dodecanol \| 66291 \| \| 4-Hepatanol, 2-methyl- \|  \| \| 3,4-Hexanediol, 2,5-dimethyl- \| 552199 \| \| n-Decanoic acid \| 2969 \| \| 4-Ethoxybenzhydrazide \| 143561 \| \| Undecanoic acid \| 8180 \| \| Tetradecanoic acid \| 11005 \| \| 9,12-Octadecadienoic acid (Z,Z)- \| 5280450 \| \| 3-Methyl-2-(2-oxopropyl)furan \| 545772 \| \| 6,8-Dodecadien-1-ol (6Z,8E) \| 5365689 \| \| 1,2-Benzenedicarboxylic acid, diisooctyl ester \| 33934 \| \| Squalene \| 638072 \| | | | | | | | | | | | | ^41^ |
| 17 | *Bauhinia variegata* (L.) Benth. | \| 2,5-Dimethyl,1 H Pyrole, \| 12265 \| \| --- \| --- \| \| 5,6,7,7a-tetrahydro-4,4 2(4H)-Benzofuranone \| 27209 \| \| Dihydroactinidiolide (Haroring agent-volatile terpene of black tea/fenugreck/ants) \| 6432173 \| \| 3,7,11-trimethyl-1-dodecanol \| 138824 \| \| Acetic acid,3,7,11,15-tetramethyl hexadecyl ester \| 54558 \| \| 1-Tetradecene \| 14260 \| \| 6 Hydroxy-4-4,7a trimethyl-5,6,7,7a Tetrahydrobenzofuran-2(4H) one \| 100332 \| \| Isopropyl Myristate \| 8042 \| \| Neophytadiene \| 10446 \| \| 2,Methyl,7-Octadecyne \| 118810 \| \| Phytol acetate \| 6428538 \| \| 1,2 Benzene dicarboxylic acid, butyl 2 methyl propyl ester \| 288813 \| \| 3,7,11,15-Tetramethyl-2-hexadecen-1-ol \| 5366244 \| \| Formic acid, 3,7,11-trimethyl-1,6,10-dodecatrien-3-yl ester \| 5363406 \| \| 2,6,10,14-Hexadecatetraen-1-ol, 3,7,11,15-tetramethyl-, acetate \| 5366012 \| \| 4,8,13-Cyclotetradecatriene-1,3-diol, 1,5,9-trimethyl-12-(1-methylethyl)- \| 5367548 \| \| Hexa decanoic acid, methyl ester \| 8181 \| \| Phytol \| 5280435 \| \| 4,8,12,16-Tetramethylheptadecan-4-olide \| 567149 \| \| 2H-Pyran-2-one, tetrahydro-6-nonyl \| 520296 \| \| Hexadecane \| 11006 \| \| 5,5-Diethylpentadecane \| 85977274 \| \| Eicosane \| 8222 \| \| 2,3-Diphenylcyclopropyl)methyl phenyl sulfoxide, trans- \| 562543 \| \| 1,2-Propanediol, 3-benzyloxy-1,2-diacetyl \| 114563 \| \| Benzonitrile, m-phenethyl \| 141877 \| \| Phthalic acid, di (2-propylpentyl) ester \| 191964 \| \| 1, 3 Bengere Oi carborglic acid, bisrethyl hexyl ester \| --- \| \| Alpha-Tocospiro-A \| 21674156 \| \| Alpha-Tocospiro-B \| 21674157 \| \| Liodo, Tricontane \| ------ \| \| 2Methyl pentacosane \| 526118 \| \| Gamma Tocopherol \| 92729 \| \| Beta Tocopherol \| 6857447 \| \| Delta Tocopherol-o-methyl \| 77299178 \| \| Cholesta-4,6 - dien - 3 - ol (3 beta) \| 14795191 \| \| Stigmast-5-en-3-ol, oleate \| 20831071 \| \| Stigmast-3, 5 diene \| 13783149 \| \| Alpha Tocopherol-beta D-mannoside \| 597057 \| \| Vitamin E \| 14985 \| \| O-methyl(+)-gamma Tocopherol \| 57978470 \| \| Ergost-5-en-3-ol (3-beta) (Campesterol) \| 6428659 \| \| Stigmasterol \| 5280794 \| \| Gamma-Sitosterol \| 457801 \| \| Beta Sitosterol \| 222284 \| \| Lup-20 (2g)-en-3-one \| 92158 \| \| Lup-20 (29)-en-3-0l, acetate \| 92157 \| \| Lupeol \| 259846 \| \| Betulin aldehyde \| 99615 \| | | | | | | | | | | | | ^42^ |
| 18 | *Glycine max* (L.) Merr. | \| Carbamide \| 1176 \| \| --- \| --- \| \| Propanal, 3-methoxy \| 137720 \| \| n-hexane \| 8058 \| \| Acetamide, oxime \| 178 \| \| 1.2-naphthalenedione, 4 chloro \| 582391 \| \| 1,3-Dioxolane-4-methanol \| 21618 \| \| 1-monolinoleoyglycerol trimethylsilyl ether \|  \| \| acetic acid \| 176 \| \| Acetic acid, 2,2-[oxybis(2,1-ethanediyloxy)]bis \| 83793 \| \| Ethyl(dimethyl)isopropoxysilane \| 554635 \| \| Silane, triethylmethoxy- \| 525763 \| \| Butanoic acid, 4,4-dithiobis[2-amino-,[S-(R*,R*)] \| 439579 \| \| 2-Pyridinecarboxylic acid \| 1018 \| \| 2-propanone, 1-(dimethylamino)- \| 84883 \| \| 2,2-bioxirane \| 92270 \| \| Cyclotrisiloxane, hexamethyl \| 10914 \| \| Pyrimidine, 2-methyl- \| 78748 \| \| L-Galactose, 6-deoxy- \| 3034656 \| \| 2-Propenamide \| 6579 \| \| Pyrazine, ethyl- \| 26331 \| \| Oxirane, 2-ethyl-2-methyl \| 121763 \| \| Butyrolactone \| 7302 \| \| 4-methyl-2-haptanol \|  \| \| 1,2-cyclopentanedione \| 566657 \| \| Pyran-4-Carboxylic acid, 4-(4-methoxyphenyl)-tetrahydro- \| 969802 \| \| 6-Oxa-bicyclo[3.1.0]hexan-3-one \| 535532 \| \| Dihydroxyacetone \| 670 \| \| Butanoic acid, 4-hydroxy- \| 10413 \| \| Propanedioic acid, Propyl- \| 12027 \| \| 1,2,3-Propanetriol \| 753 \| \| 2,4-Dihydroxy-2,5-dimethyl-3(2H)-furan-3-one \| 538757 \| \| Oxirane, [(2-propenyloxy)methyl}- \| 7838 \| \| 2H-Pyran-2,6(3H)-dione \| 574367 \| \| 1H-indazole, 4,5, 6, 7-tetrahydro \| 648852 \| \| 2-Pyrrolidinone, 1-methyl \| 13387 \| \| Benzeneacetaldehyde \| 998 \| \| 2,4,6,-Cycloheptatrien-1-one,4-methyl- \| 11007879 \| \| 2,5-Dimethyl-4-hydroxy-3(2H)-furanone \| 19309 \| \| phenol, 2-methoxy- \| 460 \| \| Formic acid, 3-methylbut-2-yl ester \| 19810797 \| \| 1-Butanol,3-methyl-,formate (isopentyl alcohol, formate) \| 8052 \| \| 1,5-Hexadien-3-ol \| 79122 \| \| Cyclopentane, (1,1-dimethylethyl)-{Tert-Butylcyclopentane} \| 520923 \| \| 4H-Pyran-4-one,3-hydroxy-2-methyl- \| 8369 \| \| 5-hepten-3-one, 5-methyl- \| 5364924 \| \| 2-acetyl-2,3,5,6-tetrahydro-1,4-thiazine \| 530434 \| \| 3,5-Dihydroxy-6-methyl-2,3-dihydro-4H-pyran-4-one \| 119838 \| \| Propanal, 2-(benzoyloxy)-,® \| 10866899 \| \| Benzoic Acid \| 243 \| \| N-aminomorpholine \| 20315 \| \| pentanoic acid, 2-isopropoxyphenyl ester \| 559066 \| \| Phenol, 4-ethenyl-, acetate \| 75821 \| \| Benzaldehyde, 3,4-dimethyl- \| 22278 \| \| Benzene, (ethenyloxy)- \| 69840 \| \| Benzofuran, 2,3-dihydro \| 2566 \| \| Benzeneacetaldehyde, 3-methyl \| 175272 \| \| 1,2-Benzenediol,3-methoxy- \| 13622 \| \| Isosorbide \| 12597 \| \| 2-Methoxy-4-vinylphenol \| 332 \| \| (p-Hydroxyphenyl)glyoxal \| 90568 \| \| 2-Acetamido-2-deoxy-d-mannolactone \| 536792 \| \| Phenol, 2,6-dimethoxy- \| 7041 \| \| 1-undecanol alcohol \| 8184 \| \| Phenol,2,6-bis(1,1-dimethylethyl)-4-methyl- \| 31404 \| \| Phenol, 2,4-bis(1,1-dimethylethyl)- \| 7311 \| \| 5-tert-Butyl-1,2,3-benzenetriol \| 597592 \| \| Benzoic acid, 4-ethoxy-, ethyl ester \| 90232 \| \| 3,4-0-Isopropylidene-d-galactose \|  \| \| 3,5-Dimethoxyacetophenone \| 95997 \| \| a-Methyl-D-mannopyranoside \| 469459093 \| \| a-D-Galactopyranoside, methyl \| 94214 \| \| 3-O-methyl-d-glucose \| 8973 \| \| 2,6-diisopropylnapthalene \| 32241 \| \| Dodecyl acrylate \| 75084 \| \| Phthalic acid,hex-3-yl-isobutyl ester \| 91719722 \| \| Hexadecanoic acid, methyl ester \| 8181 \| \| 5,8, 11-heptadecatriynoic acid methyl ester \| 582271 \| \| Phthalic acid, butyl undecyl ester \| 6423450 \| \| 1,2-benzenedicarboxylic acid,dibutyl ester \| 3026 \| \| methyl 10 trans, 12-cis-octadecadienoate \| 5471014 \| | | | | | | | | | | | | ^43^ |
| 19 | *Berberis aristata* DC. | \| n- Hexadecanoic acid \| 985 \| \| --- \| --- \| \| Linoleic acid ethyl ester \| 5282184 \| \| Benzene,1,2-dimethoxy-4-(1-propenyl)- \| 637776 \| \| Canadine \| 34458 \| \| Glycerol, tris (trimethylsilyl) ether \| 522285 \| \| Piperazine \| 4837 \| \| 1-Piperazineethanamine, 4-methyl- \| 70284 \| \| Berberine \| 2353 \| \| Taraxasterol \| 115250 \| \| Lanosterol \| 246983 \| \| β - Sitosterol \| 222284 \| \| Stigmasterol \| 5280794 \| \| Octadecanoic acid, methyl ester \| 8201 \| \| Phytol \| 5280435 \| \| 9,12,15 – Octadecatrienoic acid, methyl ester, (Z,Z,Z)- \| 5319706 \| \| 9,12- Octadecadienoic acid (Z,Z) - , methyl ester \| 5284421 \| \| Hexadecanoic acid, methyl ester \| 8181 \| \| Bicyclo [3.1.1] heptane,2,6,6- trimethyl- \| 10129 \| \| Diethyl phthalate \| 6781 \| \| 2-Pentanone,4-Hydroxy-4-methyl- \| 31256 \| \| 3-Hexen-2-one \| 5367744 \| \| Pyridine \| 1049 \| | | | | | | | | | | | | ^44^ |
| 20 | *Nigella sativa* L. | \| 5Hydroxy methyl furfural \| 237332 \| \| --- \| --- \| \| Thymoquinine \| 10281 \| \| Thymol \| 6989 \| \| 2-Isopropylidene-5-methylhex-4-enal \| 534886 \| \| Longifolene \| 289151 \| \| Phenol, 3-(1,1-dimethylethyl)-4-methoxy \| 6932 \| \| p-tert-Butyl catechol \| 7381 \| \| Tetradecanoic acid \| 11005 \| \| Tetradecanoic acid, ethyl ester \| 31283 \| \| Hexadecanoic acid, methyl ester \| 8181 \| \| n-Hexadecanoic acid \| 985 \| \| Hexadecanoic acid, ethyl ester \| 12366 \| \| 9,12-Octadecadienoic acid, methyl Ester \| 5284421 \| \| Oleic Acid \| 445639 \| \| 9,12-Octadecadienoic acid (Z,Z) \| 5280450 \| \| Eicosanoic acid \| 10467 \| \| cis-13,16-Docasadienoic acid \| 5312554 \| \| cis-11,14-Eicosadienoic acid, methyl ester \| 6430995 \| \| Methyl 19-hexacosenoate \| 91692796 \| \| Cholestan-3-ol, 2-methylene-, (3β ,5α ) \| 221122 \| | | | | | | | | | | | | ^45^ |
|  |  | Thymol | | | | | | | | | | | 6989 | ^46^ |
|  |  | 2-Isopropylidene-5-methylhex-4-enal | | | | | | | | | | | 534886 |  |
|  |  | Longifolene | | | | | | | | | | | 1796220 |  |
|  |  | Phenol, 3-(1,1-dimethylethyl)-4-methoxy | | | | | | | | | | | 6932 |  |
|  |  | p-tert-Butyl catechol | | | | | | | | | | | 7381 |  |
|  |  | Tetradecanoic acid | | | | | | | | | | | 11005 |  |
|  |  | 9,12-Octadecadienoic acid, methyl Ester | | | | | | | | | | | 5284421 |  |
|  |  | Oleic Acid | | | | | | | | | | | 445639 |  |
|  |  | 9,12-Octadecadienoic acid (Z,Z) | | | | | | | | | | | 5280450 |  |
| 21 | *Acorus calamus* L. | \| hexanal \| 6184 \| \| --- \| --- \| \| tricyclene \| 79035 \| \| α-pinene \|  \| \| camphene \| 6616 \| \| benzaldehyde \| 240 \| \| sabinene \| 18818 \| \| β-pinene \|  \| \| myrcene \| 31253 \| \| δ−3-carene \| 26049 \| \| p-cymene \| 7463 \| \| limonene \| 22311 \| \| (Z)-β-ocimene \|  \| \| (E)-β-ocimene \|  \| \| γ-terpinene \| 7461 \| \| terpinolene \| 11463 \| \| linalool \| 6549 \| \| camphor \| 2537 \| \| cis-β-terpineol \|  \| \| borneol \| 64685 \| \| terpinen-4-ol \| 11230 \| \| α-terpineol \| 17100 \| \| trans-carveol \| 94221 \| \| citronellol \| 8842 \| \| geraniol \| 637566 \| \| methyl citronellate \| 61290 \| \| bornyl acetate \| 93009 \| \| monoterpene alcohol acetate \|  \| \| α-copaene \| 19725 \| \| sesquiterpene hydrocarbon \|  \| \| β-elemene \| 6918391 \| \| 1,7-di-epi-β-cedrene \| 6431123 \| \| isocariophylene \| 5281522 \| \| β−caryophyllene \| 5281515 \| \| β-gurjunene \| 6450812 \| \| trans-α-bergamotene \| 6429302 \| \| aromadendrene \| 11095734 \| \| β-humulene \| 5318102 \| \| sesquiterpene \| 13818797 \| \| sesquiterpene \| 13818797 \| \| α-humulene \| 5281520 \| \| (Z)-methyl isoeugenol \| 1549045 \| \| β-acoradiene \| 5316209 \| \| sesquiterpene \|  \| \| ar-curcumene \| 3083834 \| \| β-selinene \| 442393 \| \| α-selinene \| 10856614 \| \| shyobunone isomer \| 91751381 \| \| sesquiterpene (β-sesquiphellandrene) \|  \| \| δ-cadinene \| 10657 \| \| 6,11-oxidoacor-4-ene \|  \| \| α-calacorene \| 12302243 \| \| germacrene B \| 5281519 \| \| β-calacorene \| 529621 \| \| (E)-nerolidol \| 5284507 \| \| spathulenol \| 92231 \| \| (Z)-isoelemicin \| 5851118 \| \| sesquiterpenoid \| 139087999 \| \| (Z)-asarone \| 5281758 \| \| elemicin \| 10248 \| \| sesquiterpenoid \| 139087999 \| \| acorenone \| 12480741 \| \| (E)-asarone \| 636822 \| \| sesquiterpenoids mixture \|  \| \| 8-α,11-elemenediol \|  \| \| acorone \| 5316254 \| \| isoacorone \| 11402120 \| | | | | | | | | | | | | ^47^ |
| 22 | *Moringa oleifera* Lam. | \| Dihydroxyacetone \| 670 \| \| --- \| --- \| \| Glycerin \| 753 \| \| Erythritol \| 222285 \| \| Monomethylmalonate \| 11857 \| \| 4,5-Diamino-6-hydroxypyrimidine \| 135436550 \| \| 4H-Pyran-4-one,2,3-dihydro-3,5-dihydroxy-6-methyl- \| 119838 \| \| Furan,2,3-dihydro-4-methyl- \| 36744 \| \| Catecholborane \| 6327445 \| \| 2-Fluoropyridine \| 9746 \| \| 1,2,3-Propanetriol,1-acetate \| 33510 \| \| 3,4-Furandiol, tetrahydro-, trans- \| 90803 \| \| 1-Nitro-.beta.-d-arabinofuranose, tetraacetate \| 536786 \| \| 1,8-Diamino-3,6-dioxaoctane \| 70248 \| \| 1,7-Diaminoheptane \| 69533 \| \| N,N-Dimethylacetamide \| 31374 \| \| Oxazolidine,2-ethyl-2-methyl- \| 278565 \| \| Heptanal \| 8130 \| \| 6-Methoxy-3-pyridazinethiol \| 5372655 \| \| 3-Piperidinol \| 23293 \| \| 1,3-Propanediol,2-ethyl-2-(hydroxymethyl)- \| 6510 \| \| Benzeneacetonitrile,4-hydroxy- \| 26548 \| \| Benzenebutanal, .gamma.,4-dimethyl- \| 583405 \| \| Formamide,N,N-dimethyl- \| 6228 \| \| d-Talonic acid lactone \| 608 \| \| Sorbitol \| 5780 \| \| Allo-Inositol \| 892 \| \| Octadecanoicacid \| 5281 \| \| 4-Allyl-3-(dimethylhydrazono)-2-methylhexane-2,5-diol \| 9601572 \| \| Benzyl .beta.-d-glucoside \| 439503 \| \| 4,6-dimethyl-2-propyl-1,3,5-dithiazinane \| 71333279 \| \| 1,3-Benzenediol,2-methyl- \| 11843 \| \| Phytol \| 5280435 \| | | | | | | | | | | | | ^48^ |
| 23 | *Withania somnifera (L.)* Dunal | \| Guanosine \| 135398635 \| \| --- \| --- \| \| Phosphoric acid, diethyl octyl ester \| 209698 \| \| Dodecanoic acid \| 3893 \| \| 1-TretradecanamineN,N-dimethyl \| 8211 \| \| Tetradecanoic acid \| 11005 \| \| Isopropyl myristate \| 8042 \| \| 2-Hexadecene,3,7,11,15-tetramethyl- \| 5366161 \| \| 2,6,10 Trimethyl, 14-Ethylene-14-pentadecne \|  \| \| 1-Decanol,2-octyl \| 3084890 \| \| 2-Hexadecen-1-ol \| 534571 \| \| 3,7,11,15-Tetramethyl-2-hexadecen-1-ol \| 5366244 \| \| 9,12,15-Octadecatrien-1-ol, (Z,Z,Z) \| 6436081 \| \| Hexadecanoic Acid \| 985 \| \| Ethyl Ester \| 54670067 \| \| 2-methyltetracosane \| 527459 \| \| Phytolisomer \|  \| \| cis,cis,cis-7,10,13-Hexadecatrienal \| 5367366 \| \| 9,12,15-Octadecatrien-1-ol \| 5367327 \| \| Docosanoic acid, ethyl ester \| 22199 \| \| 3-Cyclopentylpropionic acid \| 8818 \| \| 2H-Pyran,2-(2-heptadecynyloxy) tetrahydro- \| 544115 \| \| 2- dimethyl amino ethyl ester \| 5288826 \| \| 1-Octadecene \| 8217 \| \| Methyl 2-O-acetyl-3,4-di-o-methyl-alpha-D-xylopyranosid \|  \| \| 1,2-Benzenedicarboxylic acid \| 1017 \| \| 2,5-Cyclohexadiene-1,4-Dione \| 4650 \| \| Cyclododecanone-2-methylene \| 534631 \| \| Isopropyl linoleate \| 5352860 \| \| 2-methylhexacosane \| 150931 \| \| 2-[12-(2-oxiranyl) dodecyl] oxirane \| 543423 \| \| Squalene \| 638072 \| \| Phytol \| 5280435 \| \| Neophytadiene \| 10446 \| \| Pentatriacontane \| 12413 \| \| 3alpha,5alpha-cyclo-ergosta-7,9(11),22triene-6beta-ol \|  \| \| Retinol \| 445354 \| \| Trihydroxycholanic acid \| 69333865 \| \| Tocopherol \|  \| \| Celidoniol \| 16057860 \| \| Stigmast-5-en-3-ol \| 22012 \| \| Vitamin E \|  \| \| Ergosta-7,22-Dien-3-ol \| 125947 \| \| Pivalate \| 6919101 \| \| Propylidenecholesterol \| 5281328 \| \| Ergost-5-en-3-ol, (3.beta.)- \| 173183 \| \| Stigmasta-5, 22-Dien-3-ol \|  \| \| 24-Propylidene-(3 beta) \| 6443745 \| \| Sitostero \|  \| \| Fucosterol \| 5281328 \| | | | | | | | | | | | | ^49^ |
| 24 | *Aegle marmelos (L.)* Corrêa | \| 4H-Pyran-4-one, 2,3-dihydro-3,5-dihydroxy-6-methyl \| 15114468 \| \| --- \| --- \| \| 1-Butanol,3-methyl-,acetate \| 31276 \| \| Bicyclo[3.1.1]heptane-2,3-diol, 2,6,6-trimethyl (2,3-Pinanediol) \| 6553875 \| \| 2-Cyclohexen-1-one, 4-hydroxy-3-methyl-6-(1-methylethyl) \| 22215384 \| \| 1-Dodecanol \| 31404 \| \| Phenol, 2,6-bis(1,1-dimethylethyl)-4-methyl (BHT) \| 31404 \| \| Benzoicacid,4-ethoxy-,ethylester \| 241070998 \| \| 1-Tetradecanol,acrylate \| 8209 \| \| 1,3,4,5-Tetrahydroxycyclohexanecarboxylicacid (Quinicacid) \| 6508 \| \| Tetradecanoicacid (Myristicacid) \| 11005 \| \| 1-Heptadecanol (1-Eicosanol) \| 12404 \| \| 1,3-Cyclohexadiene,2-methyl-5-(1methylethyl)-(1-Phellandrene) \| 11605 \| \| 1,6-Octadiene,7-methyl-3-methylene (beta.-myrcene) \| 31253 \| \| alpha.-Tocopherol \| 2116 \| \| 3,7,11,15-Tetramethyl-2-hexadecen-1-ol (Phytol) \| 5280435 \| \| hexadecanoicacid,methylester (Palmiticacidmethylester) \| 985 \| \| Pentadecanoicacid \| 13849 \| \| 9-Octadecenoicacid \| 637517 \| \| Heptadecanoicacid \| 2737348 \| \| 9,12,15-Octadecatrienoicacid,methyl ester (Linolenicacid,methylester) \| 5280934 \| \| 2-Hexadecen-1-ol,3,7,11,15-tetramethyl (Phytolisomer) \| 145386 \| \| Cis-9-Hexadecenal \| 5364643 \| \| Benzene,1,2-dimethoxy-4-[[(4 methylphenyl)sulfonyl]methyl \| 592100 \| \| Cholest-5-en-3-ol(3.beta.)- \| 5997 \| \| Ergost-5-en-3-ol,(3.beta.)- \| 6428659 \| \| Stigmasta-5,22-dien-3-ol \| 5280794 \| \| Stigmast-5-en-3-ol,(3.beta.)- \| 222284 \| | | | | | | | | | | | | ^50^ |
| 25 | *Zingiber officinale* Roscoe | \| Octanal \| 454 \| \| --- \| --- \| \| Decanal \| 8175 \| \| 1,2-15,16-Diepoxyhexadecane \| 543423 \| \| Propanal,2- methyl-3-phenyl \| 5372813 \| \| 1-Heptatriacotanol \| 537071 \| \| Fenretinide \| 5288209 \| \| Folic acid \| 1.35E+08 \| \| Gingerol \| 442793 \| \| Naphthalene,decahydro-1 \| 300475 \| \| 13-Docosenamide(Z) \| 5365369 \| \| Ingol 12-acetate \| 536571 \| \| Corymbolone \| 178931 \| \| Phenol,2-methoxy-5-(1-propenyl)-,E \| 101959 \| \| Alfa.-Copaene \| 19725 \| \| Piperine \| 638024 \| \| Longipinocarveo \| 534645 \| \| Aromadendrene oxide \| 91753455 \| \| Alloaromadendrene \| 10899740 \| | | | | | | | | | | | | ^51^ |
|  |  | Glycidol | | | | | | | | | | 11164 | | ^52^ |
|  |  | 2-Aminononadecane | | | | | | | | | | 141647 | |  |
|  |  | Rimantadine | | | | | | | | | | 5071 | |  |
|  |  | Curcumene | | | | | | | | | | 92139 | |  |
|  |  | Zingiberene | | | | | | | | | | 99776 | |  |
|  |  | α-Farnesene | | | | | | | | | | 5281516 | |  |
| 26 | *Silybum marianum (L.)* Gaertn. | \| 8-Octadecenoicacid, methyl ester \| 5364422 \| \| --- \| --- \| \| Heptadecanoic acid, 14-methyl-, methyl ester, \| 17219 \| \| Benzene, 1-isocyanato-3-methoxy- \| 87843 \| \| Carbamic acid, (3-methylphenyl)-, methyl ester \| 596693 \| \| Pentadecanoic acid, 14-methyl-, methyl ester \| 21205 \| \| 11-Octadecenoic acid, methyl ester \| 74738 \| \| 2-(2-Methoxy-5-methyl-phenyl)-propanaldehyd \| 590884 \| \| Silane, (1,1-dimethylethyl)dimethyl (phenylmethoxy)- \| 596642 \| \| 2-Undecanone, 6,10-dimethyl- \| 95495 \| \| 2-Pentadecanone, 6,10,14-trimethyl- \| 10408 \| \| 7-Octadecenoic acid, methyl ester \| 5364440 \| \| Hexadecanoic acid, methyl ester \| 530322 \| \| Dibutyl phthalate \| 3026 \| \| Methyl stearate \| 8201 \| \| Tetradecanoic acid, 12-methyl-, methyl ester \| 21672 \| | | | | | | | | | | | | ^53^ |
|  |  | \| silymarin \| 5213 \| \| --- \| --- \| \| silybin \| 31553 \| \| silybin A \| 31553 \| \| silybin B \| 1548994 \| \| isosilybin A \| 11059920 \| \| isosilybin B \| 10885340 \| \| silibinin-d \| 165412574 \| \| silydianin \| 11982272 \| \| silychristin \| 4481797 \| | | | | | | | | | | | | ^54^ |
| 27 | *Capsicum annuum* L. | \| Pentadecane \| 12391 \| \| --- \| --- \| \| Heneicosane \| 12403 \| \| Methyl tetradecanoate \| 31284 \| \| Tetradecanoic acid \| 16213484 \| \| Octadecane \| 11635 \| \| 2-hydroxy-Cyclopentadecanone \| 543400 \| \| Pentadecanoic acid \| 13849 \| \| Nonadecane \| 12401 \| \| Methyl palmitoleate \| 643801 \| \| Methyl hexadecanoate \| 87370569 \| \| Palmitoleic acid \| 445638 \| \| n-Hexadecanoic acid \| 628589 \| \| 9,10-Anthracenedione \| 92021 \| \| Methyl linoleate \| 5284421 \| \| Methyl oleate \| 5364509 \| \| Oleic acid \| 445639 \| \| Ethyl oleate \| 5363269 \| \| Ethyl linolenate \| 5367460 \| \| 2-Hydroxyethyl hexadecanoate \| 20201 \| \| 1-Heneicosanol \| 85014 \| \| bis(2-ethylhexyl) hexanedioate \| 40468166 \| \| 2-hydroxyethyl octadecanoate \| 87153117 \| \| Nonivamide \| 2998 \| \| Capsaicin \| 1548943 \| \| Dihydrocapsaicin \| 107982 \| \| Homocapsaicin \| 6442566 \| \| Homodihydrocapsaicin \| 3084336 \| \| γ-Tocopherol \| 92729 \| \| DL-α-Tocopherol \| 2116 \| \| Ergost-5-en-3-ol \| 18660356 \| \| Stigmasterol \| 5280794 \| \| γ-Sitosterol \| 133082557 \| \| D-Glucuronic acid \| 94715 \| \| Citric acid \| 311 \| \| 1-Cyclohexene1-carboxylic acid \| 69470 \| \| Butanoic acid \| 16213394 \| \| Melibiose \| 11458 \| \| β-Gentiobiose \| 441422 \| | | | | | | | | | | | | ^55^ |
| 28 | *Colchicum autumnale* L. | \| Limonene \| 439250 \| \| --- \| --- \| \| cis-Linalool oxide \| 91752909 \| \| Linalool \| 443158 \| \| Terpinolene \| 11463 \| \| α-Terpineol \| 17100 \| \| Geranyl vinyl ether \| 5365842 \| \| β-Damascenone \| 32052 \| \| Hexahydrofarnesyl acetone \| 10408 \| \| Hexanal \| 10460 \| \| (E)-2-Hexenal \| 129776015 \| \| 2-Hexenal \| 5281168 \| \| Heptanal \| 8130 \| \| Octanal \| 454 \| \| 2-Hydroxy benzaldehyde \| 90659539 \| \| 3-Methyl benzaldehyde \| 12105 \| \| Nonanal \| 31289 \| \| (E,E)-2,6-Nonadienal \| 636687 \| \| 2-Nonenal \| 5283335 \| \| (E)-2-Nonenal \| 129727552 \| \| Decanal \| 8175 \| \| (E,Z)-2,4-Decadienal \| 6427087 \| \| Undecanal \| 8186 \| \| (E,E)-2,4-Decadienal \| 5283349 \| \| Hexadecanal \| 984 \| \| Heptadecanal \| 71552 \| \| Octadecanal \| 12533 \| \| Heptane \| 8900 \| \| Methyl cyclohexane \| 7962 \| \| (Z)-1,4-Dimethyl cyclooctane \| 6432051 \| \| Heptadecane \| 12398 \| \| (E)-9-Octadecene \| 5364599 \| \| 1-Nonadecene \| 29075 \| \| Nonadecane \| 12401 \| \| Eicosane \| 18936 \| \| (E)-3-Eicosene \| 5365051 \| \| Heneicosane \| 12403 \| \| Docosane \| 12405 \| \| 11-Tricosene \| 5363113 \| \| Tricosane \| 12534 \| \| Tetracosane \| 12592 \| \| 1-Hexanol \| 8103 \| \| 1-Octen-3-ol \| 18827 \| \| 1-Tetradecanol \| 8209 \| \| E-2-Octadecadecen-1-ol \| 5364416 \| \| 1-Eicosanol \| 12404 \| \| Tetradecanoic acid \| 16213484 \| \| Hexadecanoic acid \| 16212358 \| \| Linoleic acid \| 5280450 \| \| Ethanone, 1-(1-cyclohexen-1-yl)- \| 11041776 \| \| 4-Hydroxy-2-methylacetophenone \| 70133 \| \| 2-Pentyl furan \| 19602 \| | | | | | | | | | | | | ^56^ |
| 29 | *Boswellia serrata* Roxb. | \| α-Pinene \| 440968 \| \| --- \| --- \| \| p-Cymene \| 7463 \| \| d-Limonene \| 129670520 \| \| d-α-Thujene \| 2444324 \| \| α-Terpineol \| 17100 \| \| Bornyl acetate \| 6448 \| \| α-Terpinolene \| 7462 \| \| Methyl chavicol \| 70235324 \| \| α-Phellandrene \| 443160 \| \| γ-Murolene \| 12313020 \| \| γ-Cadinene \| 15094 \| \| α-Copaene \| 19725 \| \| α-Murolene \| 12306047 \| \| α-Cubebene \| 86609 \| \| δ-Cadinene \| 12306054 \| \| 3,5-Dimethoxytoluene \| 77844 \| \| Germacrene D \| 24771782 \| \| Allo-aromadendrene \| 42608158 \| \| β-Caryophyllene \| 20831623 \| \| o-Methyl anisole \| 33637 \| \| Linalool \| 443158 \| \| β-Gurjunene \| 6450812 \| \| β-Bourbonene \| 62566 \| \| Camphene \| 6616 \| \| Eucalyptol \| 2758 \| \| Valencene \| 9855795 \| \| S-cis-Sabinol \| 42626427 \| | | | | | | | | | | | | ^57^ |
| 30 | *Alstonia scholaris* (L.) R.Br. | \| Akuammicine \| 10314057 \| \| --- \| --- \| \| Akuammigine \| 1268096 \| \| Nb-Demethylechitamine \| 70698176 \| \| Akuammicine N-oxide \| 10314057 \| \| Echitamidine N-oxide \| 76334042 \| \| Scholaricine \| 50900051 \| \| Alschomine \| 165365674 \| \| Akuammidine-N-oxide \| 15558574 \| \| 6,7-seco-Angustilobine B \| 13891912 \| \| Angustilobine B \| 13891905 \| \| Angustilobine B acid \| 13891905 \| \| Akuammidine \| 15558574 \| \| Echitamine \| 124841315 \| \| Echitamidine \| 10991442 \| \| 19-Epischolaricine \| 14707744 \| \| Losbanine \| 101589339 \| \| Rhazimanine \| 6443646 \| \| Manilamine \| 163184344 \| \| Picrinine \| 5320580 \| \| 5-Methoxystrictamine \| 102004590 \| \| Picralinal \| 5320550 \| \| Picraline \| 85074732 \| \| Scholaricine \| 50900051 \| \| Tubotaiwine \| 13783720 \| \| Tubotaiwine oxide \| 431067 \| \| 19,20-(E)-Vallesamine \| 13783712 \| \| Strictamine \| 21159178 \| \| Akuammidine \| 15558574 \| \| Nareline \| 6443592 \| \| 19-E-Vallesamine \| 13783712 \| \| Alstonic acids A \| 91895416 \| \| Alstonic acids B \| 91895417 \| \| Betulin \| 72326 \| \| Betulinic acid \| 64971 \| \| β-Sitosterol \| 222284 \| \| n-Tetracosane \| 12592 \| \| Ursolic acid \| 64945 \| \| α-Amyrin acetate \| 293754 \| \| Lupeol \| 259846 \| \| Isorhamnetin \| 5281654 \| \| Isorhamnetin-3-O-β-D-galactopyranoside \| 42433476 \| \| Loganin \| 87691 \| \| Linalool \| 443158 \| \| trans-Linalool oxides \| 6432254 \| \| cis- Linalool oxides \| 91752909 \| \| 2-Phenylethyl acetate \| 7654 \| \| α-Terpineol \| 17100 \| \| Terpinen-4-ol \| 5325830 \| \| Alyxialactone \| 14194343 \| \| Kaempferol \| 5280863 \| \| Quercetin \| 5280343 \| | | | | | | | | | | | | ^58^ |
| 31 | *Podophyllum peltatum* L. | \| Podophyllotoxin \| 164791 \| \| --- \| --- \| \| 4’-Demethylpodophyllotoxin \| 118701089 \| \| Deoxypodophyllotoxin \| 345501 \| \| 4’-Demethyldeoxypodophyllotoxin \| 160705 \| \| Beta peltatin \| 92122 \| \| Alpha peltatin \| 92129 \| \| Isopicropodophyllone \| 11189106 \| | | | | | | | | | | | | ^59^ |
| 32 | *Andrographis paniculata* (Burm.f.) Nees | \| 1,4 Dichlorobenzene \| 12221302 \| \| --- \| --- \| \| 1-Dodecene \| 8183 \| \| 2-Tetradecene \| 33650 \| \| Pentadecane \| 12391 \| \| Eicosane \| 8222 \| \| Phenol \| 996 \| \| 2,4,di-tert-butylphenol \| 7311 \| \| Isobutyl pthalate \| 28813 \| \| Alpha-Hexadecene \| 12395 \| \| Diethyl pthalate \| 6781 \| \| 6,10,14,trimethyl-2-pentadecanone \| 10408 \| \| Heptadecane \| 12398 \| \| Pentadecane \| 12391 \| \| Heptacosane \| 11636 \| \| 5-Octadecene \| 5364598 \| \| Octadecane \| 11635 \| \| Neophytadiene \| 10446 \| \| 2-Pentadecanone \| 61303 \| \| Neophytadiene \| 10446 \| \| 1,2-Benzenedicarboxylic acid \| 23668795 \| \| Neophytadiene \| 10446 \| \| Pentadecanoic acid, methyl ester \| 23518 \| \| palmitic acid \| 985 \| \| hexadecoic acid \| 16212358 \| \| palmitic acid ethyl ester \| 545613 \| \| ethyl palmitate \| 12366 \| \| 9-Octadecanoic acid \| 965 \| \| oleic acid \| 445639 \| \| Octadecanoic acid \| 5282750 \| \| stearic acid \| 5281 \| \| Tricosane \| 12534 \| \| Eicosane \| 8222 \| \| Tetracosane \| 12592 \| \| Pentacosane \| 12406 \| \| Di-n-octyl pthalate \| 8346 \| \| Hexacosane \| 12407 \| \| Heneicosane \| 12403 \| \| Nonacosane \| 12401 \| | | | | | | | | | | | | ^60^ |
| 33 | *Ziziphus jujuba* Mill. | \| 2,3-Butanediol, dinitrate \| 93031 \| \| --- \| --- \| \| Hydrazine, methyl- \| 6061 \| \| Acetic acid \| 176 \| \| 2-Propanone, 1-hydroxy- \| 8299 \| \| 2-Propenoic acid, 2-hydroxyethyl ester \| 481176996 \| \| 2-Propenoic acid, methyl ester \| 481177248 \| \| 2,3-Butanedione \| 650 \| \| Propanenitrile, 3,3 -oxybis- \| 73779 \| \| 2-Furancarboxaldehyde \| 7362 \| \| Silanol, dimethyl- \| 521864 \| \| 2-Furanmethanol \| 7361 \| \| Propanoic acid, 2-oxo-, methyl ester \| 11748 \| \| 4-Cyclopentene-1,3-dione \| 70258 \| \| 2-Fluoro-2-bromo-butane \| 143456 \| \| Acetic acid, dimethoxy-, methyl ester \| 66647 \| \| 2(5H)-Furanone \| 10341 \| \| 1,2-Cyclopentanedione \| 566657 \| \| 2-Furancarboxaldehyde, 5-methyl- \| 10931539 \| \| 2,4-Dihydroxy-2,5-dimethyl-3(2H)-furan-3-one \| 538757 \| \| 2H-Pyran-2,6(3H)-dione \| 574367 \| \| 2,4(1H,3H)-Pyrimidinedione, 5-methyl- \| 96126 \| \| Methyl acetoxyacetate \| 537910 \| \| 4-Hydroxy-2,5-Dimethyl-3(2 h)-Furanone \| 19309 \| \| 2,3-Dihydro-5-hydroxy-6-methyl-4H-pyran \| 6429306 \| \| Heptanoic acid \| 8094 \| \| 4H-Pyran-4-one, 2,3-dihydro-3,5-dihydroxy6-methyl \| 119838 \| \| 4H-Pyran-4-one, 3,5-dihydroxy-2-methyl- \| 70627 \| \| 5-Formyl-2-furfurylmethanoate \| 58489131 \| \| l-Alanine, N-allyloxycarbonyl-, octyl ester \| 46780127 \| \| 1-Naphthalenol \| 23672323 \| \| 3-Buten-2-one, 3-methyl-, dimer \| 13143 \| \| Silane \| 23953 \| \| Diethanolamide lauric acid \| 24082 \| \| Undecylenic acid \| 5634 \| \| Tetradecanoic acid \| 16213484 \| \| Hexadecenoic acid, Z-11- \| 5312414 \| \| n-Hexadecanoic acid \| 628589 \| \| 9,12-Octadecadienoic acid, (Z,Z) \| 5352430 \| \| methyl santolinate \| 565394 \| \| lauric acid, (Z)- \| 3893 \| \| Octadecanoic acid \| 5282750 \| \| 5,5,6-Trimethylhept-3-en-2-one \| 5363716 \| \| 9,12-Octadecadienoic acid, (Z,Z) \| 5352430 \| \| Palmitic acid.beta.-monoglyceride \| 123409 \| \| Octadecanoic acid, 2,3-dihydroxypropyl ester \| 154734731 \| \| 13-Docosenamide, (Z)- \| 5365369 \| | | | | | | | | | | | | ^61^ |
| 34 | *Podophyllum hexandrum* Royle | \| 3-Methyldecane \| 92239 \| \| --- \| --- \| \| 2, 3-Dihydro-3, 5-dihydroxy-6-methyl-4(H)-pyran-4-one \| 119838 \| \| Tridecan \| 12388 \| \| 6-Propyltridecane \| 521567 \| \| (1-Butylhexyl)benzene \| 20657 \| \| Hexadecane \| 11006 \| \| 2-Hexadecanol \| 85779 \| \| 1-Octadecane \| 11635 \| \| Methyl 14-methylpalmitate \| 8181 \| \| Palmitic acid \| 985 \| \| 7,10-Octadecadienoic acid methyl ester \| 5365663 \| \| Cis-11-octadecenoic acid methyl ester \| 5364505 \| \| Linoleic acid ethyl ester \| 5282184 \| \| Linoleic acid \| 5280450 \| \| 2,6-Dimethoxyphenol \| 7041 \| \| Tetradecane \| 12389 \| \| 6-Propyltridecane \| 521567 \| \| Heptadecane \| 12398 \| \| Myristic acid \| 11005 \| \| henicosene \| 3015374 \| \| Abietatriene \| 6432211 \| \| Oleic acid \| 445639 \| | | | | | | | | | | | | ^62^ |
|  |  | \| Podophyllotoxin \| 164791 \| \| --- \| --- \| \| Kaempferol \| 5280863 \| \| Quercetin \| 5280343 \| \| 4-dimethylpodophyllotoxin \| 118701089 \| \| alpha-peltatin \| 92129 \| \| Epipodophyllotoxin \| 105111 \| | | | | | | | | | | | | ^62^ |
| 35 | *Betula utilis* D.Don | \| Chlorogenic acid \| 1794427 \| \| --- \| --- \| \| Caffeic acid \| 689043 \| \| Ferulic acid \| 445858 \| \| Quercetin \| 5280343 \| \| Luteolin \| 5280445 \| \| Kaempferol \| 5280863 \| \| Catechin \| 73160 \| \| Apigenin \| 5280443 \| \| Betulinic acid \| 64971 \| \| Oleanolic acid \| 10494 \| | | | | | | | | | | | | ^63^ |
|  |  | \| Oleanolic acid-3-acetate \| 6708573 \| \| --- \| --- \| \| Betulin \| 72326 \| \| Lupeol \| 259846 \| \| β-sitosterol \| 222284 \| | | | | | | | | | | | | ^64^ |
| 36 | *Panax ginseng* C.A.Mey. | \| Ginsenosides Rb1 \| 9898279 \| \| --- \| --- \| \| Ginsenosides Rb2 \| 242081755 \| \| Ginsenosides Rc \| 12855889 \| \| Ginsenosides Rd \| 11679800 \| \| Ginsenosides Rg1 \| 441923 \| \| Ginsenosides Rf \| 441922 \| \| Ginsenosides Rb3 \| 12912363 \| \| Ginsenosides F1 \| 9809542 \| \| Ginsenosides F2 \| 9918692 \| \| Ginsenosides F3 \| 46887678 \| \| Ginsenosides Rg2 \| 21599924 \| \| Ginsenosides Rg3 \| 9918693 \| \| Ginsenosides Rg5 \| 11550001 \| \| Ginsenosides Rg6 \| 91895489 \| \| Ginsenosides Rh1 \| 12855920 \| \| Ginsenosides Rh4 \| 21599928 \| \| Ginsenosides Rk1 \| 11499198 \| \| Ginsenosides Rk3 \| 75412555 \| \| Ginsenosides F4 \| 73717457 \| \| Ginsenosides Ro \| 11815492 \| \| Trilinolein \| 5322095 \| \| 1,2-dilinoleoyl-3-oleoyl-glycerol \| 97042230 \| \| Malonyl ginsenoside Rd \| 14162967 \| \| Malonyl-ginsenoside Rb1 \| 3086263 \| \| Falcarinol \| 5281149 \| \| Acetylene \| 6326 \| \| Phenylacetylene \| 10821 \| \| Panaxynol \| 5469789 \| \| Panaxydol \| 5283280 \| \| phenol A acid \| 15200541 \| | | | | | | | | | | | | ^65^ |
| 37 | *Panax pseudoginseng* Wall. | \| Protocatechuic acid \| 528594 \| \| --- \| --- \| \| Vanillic acid \| 8468 \| \| Gallic acid \| 370 \| \| 2-Coumaric acid \| 637540 \| \| Caffeic acid \| 689043 \| \| Chlorogenic acid \| 1794427 \| \| Ferulic acid \| 445858 \| \| Luteolin \| 5280445 \| \| Luteolin 7 O \| 5280637 \| \| Kaempferol \| 5280863 \| \| Quercetin \| 5280343 \| \| Myricetin \| 5281672 \| \| Hesperetin \| 72281 \| \| Daidzein \| 5281708 \| \| Sinapic acid \| 10743 \| \| Palmitic acid \| 985 \| \| Stearic acid \| 5281 \| \| Arachidic acid \| 10467 \| \| Behenic acid \| 8215 \| \| Lignoceric acid \| 11197 \| \| Palmitoleic acid \| 445638 \| \| Oleic acid \| 445639 \| \| Eicosaenoic acid \| 102044916 \| \| Linoleic acid \| 5280934 \| \| A-Linolenic acid \| 132426821 \| | | | | | | | | | | | | ^66^ |
| 38 | *Gossypium hirsutum* L. | \| Caryophyllene \| 5281515 \| \| --- \| --- \| \| 1,4,8-CYCLOUNDECATRIENE,2,6,6,9-TETRAMETHYL \| 23204 \| \| .beta.-Bisabolene \| 10104370 \| \| D-Allose \| 439507 \| \| Dodecanoicacid \| 12227349 \| \| .alpha.-D-Galactopyranoside,methyl \| 76935 \| \| Caryophylleneoxide \| 1742210 \| \| .beta.-bisabolol \| 12300146 \| \| 3-Buten-2-one,4-(4-hydroxy-2,2,6-trimethyl-7-oxabicyclo[4.1 \| 5371267 \| \| Tetradecanoicacid \| 16212357 \| \| MYRACALDEHYDE1 \| 93199 \| \| 3-Buten-2-ol,2-methyl-4-(1,3,3-trimethyl-7-oxabicyclo[4.1.0] \| 5363622 \| \| 2-HEXADECEN-1-OL,3,7,11,15-TETRAMETHYL-,[R-[R \| 145386 \| \| n-Hexadecanoicacid \| 504166 \| \| 2,6,10-Dodecatrien-1-ol,3,7,11-trimethyl \| 445070 \| \| GERANYLLINALOOLISOMER \| 5365872 \| \| 9,12,15-Octadecatrienoicacid,methylester,(Z,Z,Z)- \| 9316 \| \| Phytol \| 5280435 \| \| 9,12,15-Octadecatrienoicacid,(Z,Z,Z)- \| 860 \| \| Hexadeca-2,6,10,14-tetraen-1-ol,3,7,11,16-tetramethyl \| 5365865 \| \| cis-9-Hexadecenal \| 5364643 \| \| Cyclohexane,tetradecyl \| 15715 \| \| 4,8,12,16-Tetramethylheptadecan-4-olide \| 567149 \| \| 1,6,10,14,18,22-Tetracosahexaen-3-ol,2,6,10,15,19,23-hexa \| 168434007 \| \| Hexadecanoicacid,trimethylsilylester \| 521638 \| \| Hexadecanoicacid,2-hydroxy-1-(hydroxymethyl)ethylester \| 129853056 \| \| Bis(2-ethylhexyl)phthalate \| 8343 \| \| CYCLOHEXANONE,2,6-BIS(PHENYLMETHYLENE)- \| 1550329 \| \| 2-methylhexacosane \| 150931 \| \| 9,19-Cyclolanostan-3-ol,acetate,(3.beta.)- \| 537304 \| \| Formicacid,3,7,11-trimethyl-1,6,10-dodecatrien-3-ylester \| 5363406 \| \| 2-methylhexacosane \| 150931 \| \| Squalene \| 638072 \| \| Cyclohexane,1,2,3,5-tetraisopropyl \| 566241 \| \| Tetratetracontane \| 23494 \| \| PENTALENE,OCTAHYDRO-1-(2-OCTYLDECYL)- \| 298063 \| \| beta.-Tocopherol \| 6857447 \| \| Tetratetracontane \| 23494 \| \| 1-Triacontanol \| 68972 \| \| Cholesta-4,6-dien-3-ol,(3.beta.)- \| 53996943 \| \| Vitamin E \| 11305985 \| \| SOLANESOL \| 5477212 \| \| 1-Heptacosanol \| 74822 \| \| Stigmasterol \| 5280794 \| \| ARNOTHIANAMIDE \| 5281969 \| \| STIGMAST-5-EN-3-OL,(3.BETA.)- \| 13828710 \| \| Fucosterol \| 5281328 \| \| .beta.-Amyrin \| 73145 \| \| .alpha.-Amyrin \| 73170 \| \| Cholest-4-en-3-ol \| 91477 \| \| Phytol,acetate \| 6428538 \| \| SOLANESOL \| 5477212 \| \| 2,2,4-Trimethyl-3-(3,8,12,16-tetramethyl-heptadeca-3,7,11,15 \| 550096 \| | | | | | | | | | | | | ^67^ |
| 39 | *Passiflora caerulea* L. | \| Syringic acid \| 10742 \| \| --- \| --- \| \| Cinnamic acid \| 444539 \| \| trans-Ferulic acid \| 445858 \| \| trans-Caffeic acid \| 689043 \| \| L-Alanine \| 5950 \| \| L-Glutamic acid \| 33032 \| \| L-Methionine \| 6137 \| \| L-Histidine \| 6274 \| \| L-Tryptophan \| 6305 \| \| L-Threonine \| 6288 \| \| Norvaline \| 65098 \| \| Proline \| 145742 \| \| Vanillic acid \| 8468 \| \| L-Homoserine \| 12647 \| \| Malic acid \| 525 \| \| Succinic acid \| 1110 \| \| Citric acid \| 311 \| \| Margaric acid \| 10465 \| \| Linoleic acid \| 5280450 \| \| Lanosterol \| 246983 \| \| Cycloartenol \| 92110 \| \| Campesterol \| 173183 \| \| β-Sitosterol \| 222284 \| \| Stigmasterol \| 5280794 \| \| Arachidic acid \| 10467 \| \| Behenic acid \| 8215 \| \| Vanillic acid \| 8468 \| \| p-Coumaric acid \| 637542 \| \| L-Leucine \| 6106 \| \| L-Isoleucine \| 6306 \| \| Ascorbic acid \| 54670067 \| \| Palmitoleic acid \| 445638 \| \| Palmitic acid \| 985 \| \| Stearic acid \| 5281 \| \| Arachidic acid \| 10467 \| | | | | | | | | | | | | ^68^ |
|  |  | \| vitexin \| 5280441 \| \| --- \| --- \| \| isovitexin \| 162350 \| \| isoorientin \| 114776 \| \| saponarin \| 441381 \| \| schaftoside \| 442658 \| \| isoschaftoside \| 3084995 \| \| isovitexin-2’’-O-beta-glucoside \| 185995 \| \| vicenin-2 \| 442664 \| \| lucenin-2 \| 442615 \| \| swertisin \| 124034 \| \| Harman \| 5281404 \| \| harmol \| 68094 \| \| harmaline \| 3564 \| \| maltol \| 8369 \| \| gynocardin \| 441466 \| | | | | | | | | | | | | ^69^ |
| 40 | *Oldenlandia diffusa* (Willd.) Roxb. | \| Geniposidic acid \| 443354 \| \| --- \| --- \| \| Isovitexin \| 162350 \| \| Scandoside \| 21602023 \| \| Ursolic acid \| 64945 \| \| Oleanolic acid \| 10494 \| \| Limonene \| 22311 \| \| γ-Sitosterol \| 222284 \| \| Stigmasterol \| 5280794 \| \| Quercetin \| 5280343 \| \| Rutin \| 5280805 \| \| Amentoflavone \| 5281600 \| \| Kaempferol \| 5280863 \| \| Cyanidin \| 128861 \| \| α-Cedrol \| 6708665 \| \| Terpineol \| 17100 \| \| Beta-ionone \| 638014 \| \| Vanillic acid \| 846814542252 \| \| p-Coumaric acid \| 637542 \| \| Ferulic acid \| 445858 \| | | | | | | | | | | | | ^70^ |
|  |  | \| Esculetin \| 5281416 \| \| --- \| --- \| \| syringic acid \| 10742 \| \| melilotic acid \| 873 \| \| caffeic acids \| 134224180 \| \| p-hydroxybenzoic \| 7175 \| \| 6 alpha-hydroxygeniposide \| 6325021 \| \| Geniposide \| 107848 \| \| 10-o-benzoylscandoside methyl ester \| 15714685 \| \| asperuloside \| 84298 \| | | | | | | | | | | | | ^71^ |
| 41 | *Broussonetia papyrifera* (L.) Vent. | \| Gancaonin P \| 5481966 \| \| --- \| --- \| \| Isolicoflavonol \| 5318585 \| \| Lespedezaflavanone C \| 14542252 \| \| Vitexin \| 5280441 \| \| Apigenin \| 5280443 \| \| Pinocembrin \| 68071 \| \| Isobavachalcone \| 5281255 \| \| 4-Hydroxyisolonchocarpin \| 5321800 \| \| Luteolin \| 5280445 \| \| Cosmosiin \| 5280704 \| \| Isoorientin \| 114776 \| \| Orientin \| 5281675 \| \| 2,4,2′,4′-Tetrahydroxychalcone \| 10107266 \| \| Abyssinone II \| 10064832 \| \| Uralenol \| 5315126 \| \| Papyriflavonol A \| 10343070 \| \| Broussoflavan A \| 44257178 \| \| Dihydrokaempferol \| 122850 \| \| Quercetin \| 5280343 \| \| Bavachin \| 14236566 \| \| Isovitexin \| 162350 \| \| Broussinol \| 44257049 \| \| Sulfuretin \| 5281295 \| \| Isogemichalcone C \| 10143276 \| \| Isoliquiritigenin \| 638278 \| \| (2S)-Euchrenone \| 44593508 \| \| Broussoflavonol F \| 9866908 \| \| (2S)-Naringenin \| 439246 \| \| Broussoflavonol E \| 480828 \| \| Broussoflavonol G \| 10368916 \| | | | | | | | | | | | | ^72^ |
|  |  | \| (-)-beta-Elemene \| 6918391 \| \| --- \| --- \| \| alpha-Guaiene \| 5317844 \| \| alpha-Humulene \| 5281520 \| \| Longifolene-(v4) \| 570529 \| \| beta-Selinene \| 442393 \| \| alpha-Selinene \| 10856614 \| \| delta-Cadinene \| 441005 \| \| Humulene oxide \| 6324 \| \| exo-2-Hydroxycineole \| 529885 \| | | | | | | | | | | | | ^73^ |
| 42 | *Glycyrrhiza uralensis* Fisch. | \| liquiritin \| 503737 \| \| --- \| --- \| \| isoliquiritin \| 5318591 \| \| neoisoliquiritin \| 5320092 \| \| ononin \| 442813 \| \| glycyrrhizin \| 14982 \| \| apioside \| 5280746 \| \| isoliquiritin apioside, \| 6442433 \| \| licraside \| 14282455 \| \| araboglycyrrhizin \| 195342 \| \| 18 α-glycyrrhizin \| 14982 \| \| apioglycyrrhizin; \| 195343 \| \| Echinatin \| 6442675 \| \| licoflavone A \| 5319000 \| \| licochalcone A \| 5318998 \| \| formononetin \| 5280378 \| \| glycyrin \| 480787 \| \| licochalcone B \| 5318999 \| \| isoliquiritin \| 5318591 \| \| liquiritigenin \| 114829 \| \| licorice saponin G2 \| 14891565 \| \| isolicoflavonol \| 5318585 \| \| glycycoumarin \| 5317756 \| \| glycyrol \| 5320083 \| | | | | | | | | | | | | ^74^ |
|  |  | \| 4-hydroxybenzoic acid \| 135 \| \| --- \| --- \| \| R-(-)-Vestitol \| 182259 \| \| Isoliquiritigenin \| 638278 \| \| Medicarpin \| 336327 \| \| Tetrahydroxymethoxychalcone \| 6478421 \| \| Liquiritin apioside \| 10076238 \| | | | | | | | | | | | | ^75^ |
| 43 | *Boerhavia diffusa* L. | \| Ethylene glycol \| 81858 \| \| --- \| --- \| \| l-Valine \| 22211754 \| \| l-Alanine \| 11424808 \| \| 2-Pyrrolidinone \| 84461 \| \| l-Proline \| 13514636 \| \| l-Valine \| 11108121 \| \| l-Isoleucine \| 21632765 \| \| l-Threonine \| 91696554 \| \| Succinic acid \| 520988 \| \| Uracil \| 552702 \| \| Fumaric acid \| 5353016 \| \| l-Serine \| 90474444 \| \| D-(−)-Citramalic acid \| 526005 \| \| Malic acid \| 522155 \| \| l-Threonic acid \| 528672 \| \| l-Asparagine \| 6267 \| \| l-Glutamic acid \| 12451984 \| \| dl-Phenylalanine \| 12451977 \| \| 3,4-Dihydroxy-benzyl alcohol \| 101663520 \| \| 4-Methylcatechol \| 530364 \| \| d-Fructofuranose \| 528401 \| \| D-Pinitol \| 91750479 \| \| l-Tyrosine \| 14189425 \| \| β-d-Glucopyranose \| 13587619 \| \| d-Gluconic acid \| 13587619 \| \| Oxaloacetic acid \| 553054 \| \| d-Glucuronic acid \| 22211710 \| \| Ferulic acid \| 5379186 \| \| Ferulic acid \| 5379186 \| \| Sucrose \| 10931011 \| \| 1-Monopalmitin \| 552033 \| | | | | | | | | | | | | ^76^ |
|  |  | \| boeravinone A \| 14018346 \| \| --- \| --- \| \| boeravinone B \| 14018348 \| \| boeravinone C \| 13940641 \| \| boeravinone D \| 15081178 \| \| boeravinone G \| 11537442 \| \| boeravinone H \| 16745324 \| \| kaempferol 3-O-robinobioside \| 15944778 \| \| caffeoyltartaric acid \| 5281764 \| \| quercetin \| 5280343 \| \| kaempferol \| 5280863 \| | | | | | | | | | | | | ^77^ |
| 44 | *Vitis vinifera* L. | \| vitisinol B \| 11353654 \| \| --- \| --- \| \| viniferether A \| 101769424 \| \| viniferether B \| 101769425 \| \| ampelopsin C \| 182979 \| \| ampelopsin E \| 10439550 \| \| hopeaphenol \| 495605 \| \| quinic acid \| 6508 \| \| gallic acid \| 370 \| \| vanilic acid \| 8468 \| \| syringic acid \| 10742 \| \| caftaric acid \| 6440397 \| \| caffeic acid \| 689043 \| \| fertaric acid \| 22298372 \| \| coumarin \| 323 \| \| apigenin-7-O-glucoside \| 5280704 \| \| luteolin-7-O-glucoside \| 5280637 \| \| taxifolin \| 439533 \| \| fraxin \| 5273568 \| \| aesculin \| 5281417 \| \| fraxin \| 5273568 \| \| umbelliferone \| 5281426 \| \| dihydrochalcone \| 64802 \| \| gallocatechin \| 65084 \| \| catechin \| 9064 \| \| procyanidins \| 107876 \| \| procyanidin B1 \| 11250133 \| \| procyanidin A1 \| 9872976 \| \| quercetin-3-O-glucoside \| 5280804 \| \| myricetin \| 5281672 \| \| apigenin-7-O-glucoside \| 5280704 \| \| luteolin-7-O-glucoside \| 5280637 \| \| naringenin \| 439246 \| \| umbelliferone \| 5281426 \| \| rutin \| 5280805 \| \| protocatechuic acid \| 72 \| \| ellagic acid \| 5281855 \| | | | | | | | | | | | | ^78^ |
| 45 | *Morinda citrifolia* L. | \| Asperuloside tetraacetate \| 443336 \| \| --- \| --- \| \| Asperulosidic acid \| 11968867 \| \| Caproic acid \| 8892 \| \| Caprylic acid \| 379 \| \| Ethyl caprylate \| 7799 \| \| Hexanoic acid \| 8892 \| \| Octanoic acid \| 379 \| \| Threonine \| 6288 \| \| Tryptophan \| 6305 \| \| 3-hydroxymorindone \| 86012754 \| \| Lucidin \| 10163 \| \| Cystine \| 67678 \| \| Histidine \| 6274 \| \| Morindone \| 442756 \| \| Damnacanthal \| 2948 \| \| Rubiadin \| 124062 \| \| Alizarin \| 6293 \| \| Ricinoleic acid \| 643684 \| \| Proline \| 145742 \| \| Phenylalanine \| 6140 \| \| Quercetin 3-O-β-D-glucopyranoside \| 12304324 \| \| 1,3-dihydroxy-6-methyl anthraquinone \| 12634029 \| | | | | | | | | | | | | ^79^ |
|  |  | \| asperuloside \| 84298 \| \| --- \| --- \| \| chrysophanol \| 10208 \| \| digoxin \| 2724385 \| \| 5,6-dihydroxylucidin \| 102138995 \| \| nordamnacanthal \| 160712 \| \| quinoline \| 7047 \| \| rubiadin 1-methyl ether \| 96191 \| \| Americanin A \| 5459018 \| \| Scopoletin \| 5280460 \| \| Octanoic (caprylic) acid \| 379 \| \| Caproic acid \| 8892 \| \| Vitamin C \| 54670067 \| \| Asperulosidic acid \| 11968867 \| \| Quercetin \| 5280343 \| \| 1,3-dihydroxy-6-methyl anthraquinone \| 12634029 \| \| Niacin \| 938 \| \| 2-heptanone \| 8051 \| \| daucosterol \| 5742590 \| \| ursolic acid \| 64945 \| \| 1,5,15-trimethylmorindol \| 16203753 \| \| 3,3′-bisdemethylpinoresinol \| 46881231 \| \| americanin D \| 131752686 \| \| isoprincepin \| 44243159 \| \| deacetyl- asperulosidic acid \| 6325021 \| \| loganic acid \| 89640 \| \| 4-ethyl-2-hydroxyl-succinate \| 10130115 \| | | | | | | | | | | | | ^80^ |
| 46 | *Biophytum sensitivum* DC. | \| Amentoflavone \| 5281600 \| \| --- \| --- \| \| Isoorientin \| 114776 \| \| Orientin \| 114776 \| \| Vitexin \| 5280441 \| \| Epicatechin \| 72276 \| \| 1,2 dimethoxy benzene \| 7127 \| \| 2,methoxy 4-methyl phenol \| 7144 \| \| Linalool oxide \| 22310 \| \| Linalyl acetate \| 8294 \| \| Isophorone \| 6544 \| \| luteolin 7-methyl ether \| 5318214 \| \| 4-caffeoylquinic acid \| 9798666 \| \| 5-caffeoylquinic acid \| 9798666 \| \| 1,4-dimethoxy benzene \| 9016 \| \| (E)-linalool oxide \| 6432254 \| \| isophorone \| 6544 \| \| rhamnose \| 25310 \| \| linalyl acetate \| 8294 \| \| galacturonic acid \| 439215 \| \| epicatechin-(4β-8)-epicatechin \| 129689267 \| \| 1-octen-3-ol \| 18827 \| \| isovitexin \| 162350 \| \| isoorientin 7-O-glucoside \| 72193669 \| \| isoorientin 2”-O-rhamnoside \| 16126794 \| \| 1,2-dimethoxy benzene \| 7043 \| \| 2-methoxy-4-methyl phenol \| 7144 \| | | | | | | | | | | | | ^81^ |
|  |  | \| 2,5-Dimethyl-4-hydroxy-3(2H)-furanone \| 19309 \| \| --- \| --- \| \| Maltol \| 8369 \| \| 9,12-Octadecadienoic acid \| 3931 \| \| 9,12,15-Octadecatrienoic acid \| 5280934 \| \| 2,3- dihydroxypropyl este \| 33510 \| \| n-Hexadecanoic acid \| 985 \| \| 2- Cyclopentene -1-undecanoic acid \| 110680 \| \| Oleic acid \| 445639 \| \| 9-Octadecenal \| 5283381 \| \| 9,12- Octadecadienoic acid \| 5280450 \| \| Caffeic acid \| 689043 \| \| ferulic acid \| 445858 \| \| gallic acid \| 370 \| \| chlorogenic acid \| 1794427 \| \| rutin \| 5280805 \| | | | | | | | | | | | | ^82^ |
| 47 | *Aloe vera* (L.) Burm.f. | \| aloesin \| 160190 \| \| --- \| --- \| \| isoaloeresin D \| 76332505 \| \| aloenin B \| 6442939 \| \| Aloe emodin \| 10207 \| \| Sinapic acid \| 637775 \| \| chlorogenic acid \| 1794427 \| \| Malic acid \| 525 \| \| catechin \| 289 \| \| epicatechin \| 72276 \| \| α-tocopherol \| 3476 \| \| β-sitosterol \| 222284 \| \| benzene acetaldehyde \| 998 \| \| lauric acid \| 3893 \| \| palmitic acid \| 985 \| \| octadecanoic acid \| 5281 \| \| Squalene \| 638072 \| \| lupeol \| 259846 \| \| p-coumaroyl aloenin \| 14211227 \| \| aloveroside A \| 163064546 \| \| aloenin-2’-p-coumaroyl ester \| 14211227 \| \| stigmasterol \| 5280794 \| \| 1-dodecanol \| 8193 \| \| tricosane \| 12534 \| | | | | | | | | | | | | ^82^ |
| 48 | ***Annona crassiflora* Mart.** | \| p-Coumaric acid a \| 637542 \| \| --- \| --- \| \| Gallic acid a \| 370 \| \| Quinic acid a,b \| 6508 \| \| Ferulic acid \| 445858 \| \| Apigenin \| 5280443 \| \| Rutin \| 5280805 \| \| Kaempferol 3-O-β-D-glucoside \| 5282102 \| \| Kaempferol-3-O-rutinoside \| 5318767 \| \| Procyanidin B2 \| 122738 \| \| (-)-Epicatechin \| 72276 \| \| Xylopine \| 160503 \| \| 3′,7-Dimethoxy-3-hydroxyflavone \| 688836 \| \| 2′,5-Dimethoxyflavone \| 4231835 \| \| Romucosine \| 10734687 \| \| Annonacin \| 354398 \| | | | | | | | | | | | | ^83^ |
|  |  | \| β-pinene \| 2723720 \| \| --- \| --- \| \| β-caryophyllen \| 3860435 \| \| 2,3-butanediol \| 262 \| \| protocatechuic \| 8468 \| \| caffeic \| 689043 \| \| chlorogenic acid \| 1794427 \| \| O-α-L-arabinoside \| 5481224 \| \| trigonelline \| 5570 \| \| atherospermidine \| 77514 \| | | | | | | | | | | | | ^84^ |
| 49 | *Vaccinium macrocarpon* Aiton | \| Protocatechuic acid \| 72 \| \| --- \| --- \| \| p-Coumaric acid \| 637542 \| \| Gallic acid \| 370 \| \| Sinapinic acid \| 637775 \| \| Kaempferol \| 5280863 \| \| Quercetin \| 5280343 \| \| Syringetin \| 5281953 \| \| Quercetin-3-O-galactoside \| 5281643 \| \| 2-hydroxybenzoic acid \| 338 \| \| 3-hydroxybenzoic acid \| 7420 \| \| 4 -hydroxybenzoic acid \| 135 \| \| 2,3-dihydroxybenzoic acid \| 19 \| \| 2,5-dihydroxybenzoic acid \| 3469 \| \| 2,4-dihydroxybenzoic acid \| 1491 \| \| 3-(4-hydroxyphenyl)-propionic acid \| 10394 \| \| 3,4-dihydroxyphenylacetic acid \| 547 \| \| Hippuric acid \| 464 \| \| 3,4-dihydroxyhydrocinnamic acid \| 348154 \| \| p-Hydroxyhippuric acid \| 151012 \| \| m-Hydroxyhippuric acid \| 450268 \| \| o-Hydroxyhippuric acid \| 10253 \| \| 2-methylhippuric acid \| 91637 \| \| Quinic acid \| 6508 \| | | | | | | | | | | | | ^85^ |
| 50 | *Polygonum cuspidatum* Siebold & Zucc. | \| polydatin \| 5281718 \| \| --- \| --- \| \| Resveratrol \| 445154 \| \| Emodin \| 3220 \| \| Rhein \| 10168 \| \| 2-Ethoxystypandrone \| 71576360 \| \| 2-Methoxystypandrone \| 158739 \| \| procyanidin B \| 130556 \| \| 2, 3-O-gallate \| 65064 \| | | | | | | | | | | | | ^86^ |
| 51 | *Plumbago zeylanica* L. | \| plumbagin \| 10205 \| \| --- \| --- \| \| chitranone \| 633072 \| \| 3-biplumbagin \| 183757 \| \| chloroplumbagin \| 338719 \| \| elliptone \| 160477 \| \| seselin \| 68229 \| \| xanthyletin \| 65188 \| \| β sitosterol \| 222284 \| \| saponaretin \| 162350 \| \| isoaffinetin \| 44258239 \| \| 3-chloroplumbagin \| 338719 \| \| isozeylanone \| 100947536 \| \| zeylanone \| 5276618 \| \| droserone \| 442739 \| \| 3′-O-beta-glucopyranosyl plumbagic acid \| 6325261 \| \| 3′-o-beta-glucopyranosyl plumbagic acid \| 6325261 \| \| maritinone \| 633024 \| \| isoshinanolone \| 443777 \| | | | | | | | | | | | | ^87^ |
|  |  | \| 1-Tetradecene \| 14260 \| \| --- \| --- \| \| Phenol,2,4-bis(1,1-dimethylethyl) \| 7311 \| \| Cyclopentadecane \| 67525 \| \| 1-Octadecene \| 8217 \| \| Fenoprofen \| 3342 \| \| 3-Eicosene, (E)- \| 5365051 \| \| Methyl 7-methylhexadecanoate \| 554061 \| \| Indazol-4-one \| 20146588 \| \| 3,6,6-trimethyl-1-phthalazin-1-yl-1,5,6,7- tetrahydro- 1 \| 5293334 \| \| Methyl 11-Octadecenoate \| 5364432 \| \| 1H-Indole-2-carboxylic acid \| 72899 \| \| isopropyl ester \| 3017 \| \| Eicosanoic acid \| 10467 \| \| Anthranilic acid \| 227 \| \| 13-Docosenoic acid \| 8216 \| \| Tricosanoic acid \| 17085 \| \| 2-Methyl-7-phenylindole \| 610181 \| \| Tetracosanoic acid \| 11197 \| | | | | | | | | | | | | ^88^ |

Reference:

1. Apostolico, I. *et al.* Chemical composition, antibacterial and phytotoxic activities of Peganum harmala seed essential oils from five different localities in Northern Africa. *Molecules* **21**, (2016).

2. Faridi, P., Ghasemi, Y. & Mohagheghzadeh, A. Chemical composition of Peganum harmala smoke and volatile oil. *Journal of essential oil bearing plants* **16**, 469–473 (2013).

3. Mallmann, C. A., Brugnari, T., Abreu Filho, B. A. de, Mikcha, J. M. G. & Machinski, M. Curcuma longa L. essential oil composition, antioxidant effect, and effect on Fusarium verticillioides and fumonisin production. *Food Control* **73**, 806–813 (2017).

4. Araújo, C. & Leon, L. L. *Biological Activities of Curcuma Longa L*. *Mem Inst Oswaldo Cruz* vol. 96 (2001).

5. Naz, S., Ilyas, S., Parveen, Z. & Javed, S. Chemical analysis of essential oils from turmeric (Curcuma longa) rhizome through GC-MS. *Asian Journal of Chemistry* **22**, 3153 (2010).

6. Kattel, A. & Maga, J. A. *Volatile Compounds from Dried Jimbu (Allium Wallichii)*. (1995).

7. Bhandari, J., Muhammad, B., Thapa, P. & Shrestha, B. G. Study of phytochemical, anti-microbial, anti-oxidant, and anti-cancer properties of Allium wallichii. *BMC Complement Altern Med* **17**, 102 (2017).

8. Vidic, D., Čopra-Janićijević, A., Miloš, M. & Maksimović, M. Effects of Different Methods of Isolation on Volatile Composition of Artemisia annua L. *Int J Anal Chem* **2018**, (2018).

9. Woerdenbag, H. J. *et al.* Volatile constituents of Artemisia annua L.(Asteraceae). *Flavour Fragr J* **8**, 131–137 (1993).

10. Lin, J., Dai, Y., Guo, Y. N., Xu, H. R. & Wang, X. C. Volatile profile analysis and quality prediction of Longjing tea (Camellia sinensis) by HS-SPME/GC-MS. *J Zhejiang Univ Sci B* **13**, 972–980 (2012).

11. Hasan, M. R. *et al.* Antioxidant activity study and GC-MS profiling of Camellia sinensis Linn. *Heliyon* **10**, (2024).

12. Tian, X. *et al.* Chemical characterization of main bioactive constituents in Paeonia ostii seed meal and GC-MS analysis of seed oil. *J Food Biochem* **44**, (2020).

13. Yang, X. *et al.* Chemical profile and antioxidant activity of the oil from peony seeds (Paeonia suffruticosa Andr.). *Oxid Med Cell Longev* **2017**, 9164905 (2017).

14. Chaudhary, A., Sharma, S., Mittal, A., Gupta, S. & Dua, A. Phytochemical and antioxidant profiling of Ocimum sanctum. *J Food Sci Technol* **57**, 3852–3863 (2020).

15. Joshi, S. & Karna, A. K. ANALYSIS OF PHYTOCONSTIUENTS AND CYTOTOXIC ACTIVITIES OF DIFFERENT PARTS OF OCIMUM SANCTUM. *Int J Appl Sci Biotechnol* **1**, (2013).

16. Borah, R. & Biswas, S. P. Tulsi (Ocimum sanctum), excellent source of phytochemicals. *International Journal of Environment, Agriculture and Biotechnology* **3**, 265258 (2018).

17. Chandra, S. *et al.* Scientific evidences of anticancer potential of medicinal plants. *Food Chemistry Advances* vol. 2 Preprint at https://doi.org/10.1016/j.focha.2023.100239 (2023).

18. Al-Tai, A. A. & Al-Mayyahi, T. F. A chemical study by using GC-Mass spectrometry of the peel and seeds of Punica Granatum L. plant. *Systematic Reviews in Pharmacy* **12**, 1414–1421 (2021).

19. Occhiuto, F. *et al.* The Isoflavones Mixture from Trifolium pratense L. Protects HCN 1-A Neurons from Oxidative Stress. *Phytother. Res* **23**, 192–196 (2009).

20. Kalkunte, S. S. *et al.* Antidepressant and antistress activity of GC‐MS characterized lipophilic extracts of Ginkgo biloba leaves. *Phytotherapy Research: An International Journal Devoted to Pharmacological and Toxicological Evaluation of Natural Product Derivatives* **21**, 1061–1065 (2007).

21. Prakash, O. *et al.* A panoramic view on phytochemical, nutritional, and therapeutic attributes of Ziziphus mauritiana Lam.: A comprehensive review. *Phytotherapy Research* vol. 35 63–77 Preprint at https://doi.org/10.1002/ptr.6769 (2021).

22. Kushwaha, P. *et al.* GC-MS ANALYSIS OF BIO-ACTIVE COMPOUNDS IN METHANOLIC EXTRACT OF ZIZIPHUS MAURITIANA FRUIT. *Article in International Journal of Pharmaceutical Sciences and Research* **10**, (2019).

23. Kushwaha, P., Yadav, S. S., Singh, V. & Dwivedi, L. K. GC-MS analysis of bio-active compounds in methanolic extract of Ziziphus mauritiana fruit. *Int J Pharm Sci Res* **10**, 2911–2916 (2019).

24. Jasim, H., Hussein, A. O., Hameed, I. H. & Kareem, M. A. Characterization of alkaloid constitution and evaluation of antimicrobial activity of solanum nigrum using gas chromatography mass spectrometry (GC-MS). *Journal of Pharmacognosy and Phytotherapy* **7**, 57–73 (2015).

25. Khan, H. J. *et al.* Identification of Anticancer and Antioxidant phytoconstituents from chloroform fraction of Solanum nigrum L. berries using GC-MS/MS analysis. *Indian J Exp Biol* **54**, 774–782 (2016).

26. Chandra Kala, S. & Ammani, K. *GC-MS ANALYSIS OF BIOACTIVE COMPOUNDS IN WILD LEAF AND CALLUS EXTRACTS OF BIOPHYTUM SENSITIVUM (L)*. vol. 8 www.ijrpc.com.

27. Basappa, G. *et al.* Chemical composition, biological properties of Anisomeles indica Kuntze essential oil. *Ind Crops Prod* **77**, 89–96 (2015).

28. Batish, D. R., Singh, H. P., Kaur, M., Kohli, R. K. & Singh, S. Chemical characterization and phytotoxicity of volatile essential oil from leaves of Anisomeles indica (Lamiaceae). *Biochem Syst Ecol* **41**, 104–109 (2012).

29. Rao, Y. K., Fang, S. H., Hsieh, S. C., Yeh, T. H. & Tzeng, Y. M. The constituents of Anisomeles indica and their anti-inflammatory activities. *J Ethnopharmacol* **121**, 292–296 (2009).

30. Abubaker, M. A., Mohammed, A. A. A., Farah, A. A. M. & Zhang, J. Phytochemical screening by using GC-MS and FTIR spectrum analysis of fixed oil from Sudanese Ziziphus spina Christi seeds. *Eurasian Chemical Communications* **3**, 244–256 (2021).

31. Ads, E. N., Rajendrasozhan, S., Hassan, S. I., Sharawy, S. M. S. & Humaidi, J. R. Phytochemical screening of different organic crude extracts from the stem bark of Ziziphus spina-christi (L.). *Biomedical Research (India)* **29**, 1645–1652 (2018).

32. Asgarpanah, J. & Haghighat, E. Phytochemistry and pharmacologic properties of Ziziphus spina christi (L.) Willd. *Afr J Pharm Pharmacol* **6**, 2332–2339 (2012).

33. Sharma, R. *et al.* Glycyrrhiza glabra extract and quercetin reverses cisplatin resistance in triple-negative MDA-MB-468 breast cancer cells via inhibition of cytochrome P450 1B1 enzyme. *Bioorg Med Chem Lett* **27**, 5400–5403 (2017).

34. Batiha, G. E. S., Beshbishy, A. M., El-Mleeh, A., Abdel-Daim, M. M. & Devkota, H. P. Traditional uses, bioactive chemical constituents, and pharmacological and toxicological activities of Glycyrrhiza glabra L. (fabaceae). *Biomolecules* vol. 10 Preprint at https://doi.org/10.3390/biom10030352 (2020).

35. Chouitah, O., Meddah, B., Aoues, A. & Sonnet, P. Chemical composition and antimicrobial activities of the essential oil from glycyrrhiza glabra leaves. *Journal of Essential Oil-Bearing Plants* **14**, 284–288 (2011).

36. Miyazawa, M. & Kameoka, H. *Volatile Flavour Components of Glycyrrhizae Radix (Glycyrrhiza Glabva L. Var. G2andulgera Regel et Herder) from China*. *FLAVOUR AND FRAGRANCE JOURNAL* vol. 5 (1990).

37. Venkatesan, A., Kathirvel, A., Prakash, S. & Sujatha, V. Antioxidant, Antibacterial Activities and Identification of Bioactive Compounds from Terminalia chebula Bark Extracts. *Free Radicals and Antioxidants* **7**, 43–49 (2016).

38. Al-Mahrami, N. *et al.* In-silico and in-vitro studies to identify potential inhibitors of SARS-CoV-2 spike protein from Omani medicinal plants. *Heliyon* **10**, (2024).

39. Jayakumar, S., Vijayaraghavan, R., Saikarthik, J., Ilango, S. & Vijayakumar, J. PHYTOCHEMICAL ANALYSIS OF METHANOLIC EXTRACT OF SEEDS OF MUCUNA PRURIENS BY GAS CHROMATOGRAPHY MASS SPECTROMETRY. *Int J Pharm Sci Res* **8**, 2916–2921 (2017).

40. Sweetlin, G. P. & Daniel, R. R. *DETERMINATION OF BIOACTIVE COPMOUNDS IN ETHANOLIC EXTRACT OF CALLUS DERIVED FROM Mucuna Pruriens USING GAS CHROMATOGRAPHY AND MASS SPECTROSCOPIC TECHNIQUE*. *Journal of Natural Remedies* vol. 21.

41. Bhaskar, A. & Gopalakrishnan, V. *Phytochemical Evaluation by GC-MS and Antihyperglycemic Activity of Mucuna Pruriens on Streptozotocin Induced Diabetes in Rats*. *Article in Journal of Chemical and Pharmaceutical Research* www.jocpr.com (2011).

42. More-Adate, P., Lokhande, K. B., Swamy, K. V., Nagar, S. & Baheti, A. GC-MS profiling of Bauhinia variegata major phytoconstituents with computational identification of potential lead inhibitors of SARS-CoV-2 Mpro. *Comput Biol Med* **147**, (2022).

43. Alghamdi, S. S. *et al.* Comparative phytochemical profiling of different soybean (Glycine max (L.) Merr) genotypes using GC–MS. *Saudi J Biol Sci* **25**, 15–21 (2018).

44. Potdar, D., Hirwani, R. R. & Dhulap, S. Phyto-chemical and pharmacological applications of Berberis aristata. *Fitoterapia* vol. 83 817–830 Preprint at https://doi.org/10.1016/j.fitote.2012.04.012 (2012).

45. Nivetha, K. & Prasanna, G. International Journal of Advanced Research in Biological Sciences GC-MS and FT-IR Analysis of Nigella sativa L. Seeds. *Int. J. Adv. Res. Biol. Sci* **3**, 45–54 (2016).

46. Saleh, F. A., El-Darra, N., Raafat, K. & El Ghazzawi, I. Phytochemical analysis of Nigella sativa L. Utilizing GC-MS exploring its antimicrobial effects against multidrug-resistant bacteria. *Pharmacognosy Journal* **10**, (2018).

47. Wilczewska, A. Z. *et al.* Comparison of volatile constituents of acorus calamus and asarum europaeum obtained by different techniques. *Journal of Essential Oil Research* **20**, 390–395 (2008).

48. Bhalla, N., Ingle, N., Patri, S. V. & Haranath, D. Phytochemical analysis of Moringa Oleifera leaves extracts by GC-MS and free radical scavenging potency for industrial applications. *Saudi J Biol Sci* **28**, 6915–6928 (2021).

49. Rautela, I. *et al.* COMPARATIVE GC-MS ANALYSIS OF LEAF AND ROOT EXTRACT OF MEDICINAL PLANT WITHANIA SOMNIFERA *Corresponding Author. *Indra et al. World Journal of Pharmaceutical Research World Journal of Pharmaceutical Research SJIF Impact Factor* **7**, 956–972 (2018).

50. Mujeeb, F., Bajpai, P. & Pathak, N. Phytochemical evaluation, antimicrobial activity, and determination of bioactive components from leaves of aegle marmelos. *Biomed Res Int* **2014**, (2014).

51. Shareef, H. K., Muhammed, H. J., Hussein, H. M. & Hameed, I. H. Antibacterial effect of ginger (zingiber officinale) roscoe and bioactive chemical analysis using gas chromatography mass spectrum. *Oriental Journal of Chemistry* **32**, 817–837 (2016).

52. Javeed, A. *et al.* Comparative Assessment of Phytoconstituents, Antioxidant Activity and Chemical Analysis of Different Parts of Milk Thistle Silybum marianum L. *Molecules* **27**, (2022).

53. Emadi, S. A., Rahbardar, M. G., Mehri, S. & Hosseinzadeh, H. A review of therapeutic potentials of milk thistle (Silybum marianum L.) and its main constituent, silymarin, on cancer, and their related patents. *Iranian Journal of Basic Medical Sciences* vol. 25 1166–1176 Preprint at https://doi.org/10.22038/IJBMS.2022.63200.13961 (2022).

54. Aranha, B. C., Hoffmann, J. F., Barbieri, R. L., Rombaldi, C. V. & Chaves, F. C. Untargeted Metabolomic Analysis of Capsicum spp. by GC–MS. *Phytochemical Analysis* **28**, 439–447 (2017).

55. Baltacı, C., Öz, M., Fidan, M. S., Üçüncü, O. & Karataş, Ş. M. Chemical composition, antioxidant and antimicrobial activity of Colchicum speciosum Steven growing in Türkiye. *Pak J Agric Sci* **59**, 729–736 (2022).

56. Sharma, A. & Gajbhiye, V. *Phytochemical and Pharmacological Investigations on Boswellia Serrata*. www.phcog.net (2014).

57. Khyade, M. S., Kasote, D. M. & Vaikos, N. P. Alstonia scholaris (L.) R. Br. and Alstonia macrophylla Wall. ex G. Don: A comparative review on traditional uses, phytochemistry and pharmacology. *Journal of Ethnopharmacology* vol. 153 1–18 Preprint at https://doi.org/10.1016/j.jep.2014.01.025 (2014).

58. Jackson, D. E. & Dewick, P. M. *ARYLTETRALIN LIGNANS FROM PODOPH YLLUM HEXANDRUM AND PODOPHYLLUMPELTATUM*. vol. 23 (1984).

59. Roy, S., Rao, K., Bhuvaneswari, C., Giri, A. & Mangamoori, L. N. Phytochemical analysis of Andrographis paniculata extract and its antimicrobial activity. *World J Microbiol Biotechnol* **26**, 85–91 (2010).

60. Dilek Tepe, H. & Doyuk, F. Determination of Phytochemical Content by Chromatographic Methods and Antioxidant Capacity in Methanolic Extract of Jujube (Zizyphus jujuba Mill.) and Oleaster (Elaeagnus angustifolia L.). *International Journal of Fruit Science* **20**, S1876–S1890 (2020).

61. Jiu, X. *et al.* Dormancy release of seeds of Podophyllum hexandrum Royle accompanied by changes in phytochemicals and inorganic elements. *PLoS One* **18**, (2023).

62. Mishra, T. *et al.* Phytochemical profiling of the stem bark of Betula utilis from different geographical regions of India using UHPLC-ESI-MS/MS. *Analytical Science Advances* **2**, 497–504 (2021).

63. Verma, D., Ajgaonkar, S., Sahu, N., Rane, M. & Teli, N. Pharmacological and Phytochemical Properties of Betula utilis: An Overview.

64. Yang, Y. *et al.* Phytochemical analysis of Panax species: a review. *Journal of Ginseng Research* vol. 45 1–21 Preprint at https://doi.org/10.1016/j.jgr.2019.12.009 (2021).

65. Paul, D. *et al.* Phytochemical, nutritional and antioxidant potential of Panax bipinnatifidus and Panax pseudoginseng: A study of two underutilized and neglected species from the Eastern Himalayan region of India. *South African Journal of Botany* **149**, 837–852 (2022).

66. Kanipandian, N., Li, D. & Kannan, S. Induction of intrinsic apoptotic signaling pathway in A549 lung cancer cells using silver nanoparticles from Gossypium hirsutum and evaluation of in vivo toxicity. *Biotechnology Reports* **23**, (2019).

67. Gerasimova, A. *et al.* Metabolic Profile of Leaves and Pulp of Passiflora caerulea L. (Bulgaria) and Their Biological Activities. *Plants* **13**, (2024).

68. Sindhura, L. & Bobby, M. N. Phytochemical Profiles, Antioxidant, Antimicrobial and Cytotoxic cell lines activity of Passiflora caerulea L. *Biomedical and Pharmacology Journal* **15**, 2365–2379 (2022).

69. Al-Shuhaib, M. B. S. & Al-Shuhaib, J. M. B. Phytochemistry, pharmacology, and medical uses of Oldenlandia (family Rubaceae): a review. *Naunyn-Schmiedeberg’s Archives of Pharmacology* vol. 397 2021–2053 Preprint at https://doi.org/10.1007/s00210-023-02756-3 (2024).

70. Kaur, J., Konar, A., Chatterjee, R. & Singh, S. Review on the anti-cancerous properties of Oldenlandia diffusa Roxb. *International Journal of Pharmacognosy and Pharmaceutical Sciences* **5**, 01–07 (2023).

71. Chen, Y. *et al.* The Genus Broussonetia: An Updated Review of Phytochemistry, Pharmacology and Applications. *Molecules* vol. 27 Preprint at https://doi.org/10.3390/molecules27165344 (2022).

72. Kumar, N. N., Naveen Kumar, N., Ramakrishnaiah, H., Krishna, V. & Deepalakshmi, A. P. GC-MS ANALYSIS AND ANTIMICROBIAL ACTIVITY OF SEED OIL OF BROUSSONETIA PAPYRIFERA (L.) VENT. *Article in International Journal of Pharmaceutical Sciences and Research* **6**, 3954 (2015).

73. Sharifi-Rad, J. *et al.* Glycyrrhiza Genus: Enlightening Phytochemical Components for Pharmacological and Health-Promoting Abilities. *Oxidative Medicine and Cellular Longevity* vol. 2021 Preprint at https://doi.org/10.1155/2021/7571132 (2021).

74. Lee, E. J. *et al.* Isolation and characterization of compounds from glycyrrhiza uralensis as therapeutic agents for the muscle disorders. *Int J Mol Sci* **22**, 1–20 (2021).

75. Juneja, K. *et al.* Metabolite profiling and wound-healing activity of Boerhavia diffusa leaf extracts using in vitro and in vivo models. *J Tradit Complement Med* **10**, 52–59 (2020).

76. Thirunavoukkarasu, M. & Nayak, P. *A Review of the Plant Boerhaavia Diffusa: Its Chemistry, Pharmacology and Therapeutical Potential*. *The Journal of Phytopharmacology* vol. 5 www.phytopharmajournal.com (2016).

77. Insanu, M., Karimah, H., Pramastya, H. & Fidrianny, I. Phytochemical compounds and pharmacological activities of vitis vinifera L.: An updated review. *Biointerface Research in Applied Chemistry* vol. 11 13829–13849 Preprint at https://doi.org/10.33263/BRIAC115.1382913849 (2021).

78. Krishnaiah, D., Nithyanandam, R. & Sarbatly, R. Phytochemical Constituents and Activities of Morinda citrifolia L. in *Phytochemicals - A Global Perspective of Their Role in Nutrition and Health* (InTech, 2012). doi:10.5772/26094.

79. Abou Assi, R. *et al.* Morinda citrifolia (Noni): A comprehensive review on its industrial uses, pharmacological activities, and clinical trials. *Arabian Journal of Chemistry* vol. 10 691–707 Preprint at https://doi.org/10.1016/j.arabjc.2015.06.018 (2017).

80. Bharati, A. C. & Sahu, A. N. Ethnobotany, phytochemistry and pharmacology of Biophytum sensitivum DC. *Pharmacognosy Reviews* vol. 6 68–73 Preprint at https://doi.org/10.4103/0973-7847.95893 (2012).

81. Chandra Kala, S. & Ammani, K. *GC-MS ANALYSIS OF BIOACTIVE COMPOUNDS IN WILD LEAF AND CALLUS EXTRACTS OF BIOPHYTUM SENSITIVUM (L)*. vol. 8 www.ijrpc.com.

82. Nalimu, F., Oloro, J., Kahwa, I. & Ogwang, P. E. Review on the phytochemistry and toxicological profiles of Aloe vera and Aloe ferox. *Futur J Pharm Sci* **7**, (2021).

83. Carvalho, N. C. C. *et al.* Phytochemical Analysis of the Fruit Pulp Extracts from Annona crassiflora Mart. and Evaluation of Their Antioxidant and Antiproliferative Activities. *Foods* **11**, (2022).

84. Arruda, H. S. & Pastore, G. M. Araticum (Annona crassiflora Mart.) as a source of nutrients and bioactive compounds for food and non-food purposes: A comprehensive review. *Food Research International* vol. 123 450–480 Preprint at https://doi.org/10.1016/j.foodres.2019.05.011 (2019).

85. Baron, G. *et al.* Profiling Vaccinium macrocarpon components and metabolites in human urine and the urine ex-vivo effect on Candida albicans adhesion and biofilm-formation. *Biochem Pharmacol* **173**, (2020).

86. Ke, J. *et al.* Advances for pharmacological activities of Polygonum cuspidatum - A review. *Pharmaceutical Biology* vol. 61 177–188 Preprint at https://doi.org/10.1080/13880209.2022.2158349 (2023).

87. Shukla, B., Saxena, S., Usmani, S. & Kushwaha, P. Phytochemistry and pharmacological studies of Plumbago zeylanica L.: a medicinal plant review. *Clinical Phytoscience* **7**, (2021).

88. Adetiloye, I. S. *et al.* Agronomic Potential and Genetic Diversity of 43 Accession of Tropical Soybean (Glycine max (L) Merrill Article in. *International Journal of Plant Research* **5**, 1756–1761 (2020).
