# Supplementary material for: Uralenol, Glycyrol, and Abyssinone II as potent inhibitors of fibroblast growth factor receptor 2 from anti-cancer plants: A deep learning and molecular dynamics approach
Source: PLoS One. 2026 Jan 30;21(1):e0341498. doi: 10.1371/journal.pone.0341498 (PMC12857956; doi:10.1371/journal.pone.0341498)
Supplement: Attachment — Submitted filename: Peer Review Report.docx [file pone.0341498.s002.docx]

Peer Review Report

To authers:

1. Title and Abstract

The title is clear, concise, and appropriately reflects the topic and objective of the research (page 1). The abstract is comprehensive, including the aim, methods, key results, and conclusions; it effectively summarizes the main points (page 2).The term "promising" in the abstract could be more cautiously worded, specifying the preliminary nature of the findings.

2. Introduction

The scientific rationale is well presented, highlighting the importance of FGFR2 in several cancers (pages 2 to 4). A thorough review of prior studies and the role of FGFR2 is provided. Some parts of the introduction are somewhat lengthy and repetitive; for example, detailed signaling pathway descriptions could be more concise.

3. Materials and Methods

Detailed and clear descriptions of the 3D structure preparation of FGFR2 and ligand preparations are provided (pages 5 to 7). Software and parameters used are well-documented. The docking process using AutoDock Vina is appropriately executed. Selection of compounds based on binding energy and detailed interaction descriptions are thorough. More explicit comparison of docking results with reference drugs would strengthen validation.

Use of RNN and CHEMBL4142 dataset for predicting pIC50 is well described. Model performance metrics (R²=0.68, MSE, RMSE) are satisfactorily reported (page 11). Further discussion on the model limitations and their impact on results is recommended.

Utilization of three reputable servers to predict ADMET properties is provided, with clear explanation of physicochemical properties, absorption, and toxicity in Tables 2 and 3 (pages 16 to 18). Some ADME/T specialized terms could be briefly explained for a broader readership.

In Molecular Dynamics Simulation (MD), a 100 ns simulation with thorough details on parameters and methodology is performed. System preparation and computational parameters are comprehensively documented (pages 7 to 10). Adding more comparative graphs within the text might assist reader understanding.

4. Results

Clear ranking of top compounds with binding energies and molecular interaction visuals (Tables 1, Figures 1 and 2). Presentation of deep learning results, ADME/T analysis, and MD simulation data with RMSD, RMSF, Rg, SASA, and PCA plots (Figures 3 to 5). Well-interpretated results. More extensive comparison with similar studies would enhance the novelty and significance context.

5. Discussion

The importance of FGFR2 is adequately discussed, including up-to-date therapeutic perspectives (pages 21 to 27). Limitations are transparently acknowledged, particularly on absence of experimental validation and reliance on computational predictions. More concrete discussion on the potential clinical applications and recommended future experimental directions are encouraged.

6. Conclusion

Conclusions are clear, justified by key findings. Emphasis on the need for experimental validation for clinical progression is appropriate (pages 27 to 29).

7. Writing Suggestions:

Long sentences in “Introduction” could be divided into shorter ones to improve readability.

8. Ethical Considerations and Data Availability

Explicit clarification of ethical approval status (especially if no human/animal data used) would improve transparency.

Overall, this article presents a scientifically sound, well-documented, and advanced computational study of FGFR2 inhibitors. With minor editorial revisions, it is suitable for publication in PLOS ONE and will contribute valuable insight to the field.
