# Supplementary material for: Uralenol, Glycyrol, and Abyssinone II as potent inhibitors of fibroblast growth factor receptor 2 from anti-cancer plants: A deep learning and molecular dynamics approach
Source: PLoS One. 2026 Jan 30;21(1):e0341498. doi: 10.1371/journal.pone.0341498 (PMC12857956; doi:10.1371/journal.pone.0341498)
Supplement: Attachment — Submitted filename: Response to Reviewers.docx [file pone.0341498.s003.docx]

**Cover Letter	Sep 22, 2025**

**Ref.:** PONE-D-25-37887

**Title:** Uralenol, Glycyrol, and Abyssinone II as potent inhibitors of Fibroblast Growth Factor Receptor 2 from anti-cancer plants: a deep learning and molecular dynamics approach

**Journal:** Plos One

Dear editor and reviewer,

Thank you for reviewing and handling our manuscript (MS). We are sincerely grateful for your careful and constructive comments, as well as your valuable suggestions, which have greatly strengthened the MS. According to your suggestions and recommendations, we have thoroughly revised the MS. All amendments were made point by point in response to your suggestions, and the revised sections have been highlighted in RED.

Thank you and best regards.

Yours Sincerely and Respectfully

Prof. Mohammad Nurul Matin

Department of Genetic Engineering and Biotechnology

University of Rajshahi, Rajshahi-6205, Bangladesh

**Point-by-Point Response to Editorial Comments**

**Editorial comments**

Dear Dr. Matin,

Thank you for submitting your manuscript to PLOS ONE. After careful consideration, we feel that it has merit but does not fully meet PLOS ONE’s publication criteria as it currently stands. Therefore, we invite you to submit a revised version of the manuscript that addresses the points raised during the review process.

The referees raised important questions about the manuscript, so the authors must provide a point-by-point response to each question from all the referees.

Please submit your revised manuscript by Oct 18 2025 11:59PM. If you will need more time than this to complete your revisions, please reply to this message or contact the journal office at [plosone@plos.org](mailto:plosone@plos.org). When you're ready to submit your revision, log on to <https://www.editorialmanager.com/pone/> and select the 'Submissions Needing Revision' folder to locate your manuscript file.

Please include the following items when submitting your revised manuscript:

- A rebuttal letter that responds to each point raised by the academic editor and reviewer(s). You should upload this letter as a separate file labeled 'Response to Reviewers'.
- A marked-up copy of your manuscript that highlights changes made to the original version. You should upload this as a separate file labeled 'Revised Manuscript with Track Changes'.
- An unmarked version of your revised paper without tracked changes. You should upload this as a separate file labeled 'Manuscript'.

If you would like to make changes to your financial disclosure, please include your updated statement in your cover letter. Guidelines for resubmitting your figure files are available below the reviewer comments at the end of this letter.

We look forward to receiving your revised manuscript.

Kind regards,

Jorddy Neves Cruz
Academic Editor, PLOS ONE

**Our Response:** We would like to express our gratitude for your evaluation, cooperative comments, and careful consideration of our work with the decision “submission of revised version”. Significant revisions with corresponding corrections addressing comments and suggestions from the editor and reviewers have been incorporated into the substantially improved MS. Moreover, we have provided a point-by-point response to each question from all the referees. All modifications to the MS are highlighted in red, so that any changes can be easily reviewed by editors and reviewers.

We have carefully followed the journal guidelines in this revised version. Necessary changes have been introduced throughout the text. We have submitted the MS within the mentioned deadline. While submitting the revised MS, we have uploaded all the necessary item files labeled with:

- 'Response to Reviewers’
- 'Revised Manuscript with Track Changes’
- unmarked manuscript labeled ‘Manuscript’

Also, we have followed the guidelines for resubmitting figure files.

**Journal Requirements:**

When submitting your revision, we need you to address these additional requirements.

1. Please ensure that your manuscript meets PLOS ONE's style requirements, including those for file naming. The PLOS ONE style templates can be found at

<https://journals.plos.org/plosone/s/file?id=wjVg/PLOSOne_formatting_sample_main_body.pdf> and

[https://journals.plos.org/plosone/s/file?id=ba62/PLOSOne_formatting_sample_title_authors_affiliations.pdf](https://track.editorialmanager.com/CL0/https:%2F%2Fjournals.plos.org%2Fplosone%2Fs%2Ffile%3Fid=ba62%2FPLOSOne_formatting_sample_title_authors_affiliations.pdf/1/010f01990ddef1e3-15882ca2-c8c0-452f-958a-6387a569aba0-000000/3NM2jSJF0lLQlMOPShlyBjOMeCPLp0fSlUcri394WHI=227)

2. Please note that PLOS One has specific guidelines on code sharing for submissions in which author-generated code underpins the findings in the manuscript. In these cases, we expect all author-generated code to be made available without restrictions upon publication of the work. Please review our guidelines at <https://journals.plos.org/plosone/s/materials-and-software-sharing#loc-sharing-code> and ensure that your code is shared in a way that follows best practice and facilitates reproducibility and reuse.

3. Please include a separate caption for each figure in your manuscript.

4. If the reviewer comments include a recommendation to cite specific previously published works, please review and evaluate these publications to determine whether they are relevant and should be cited. There is no requirement to cite these works unless the editor has indicated otherwise. 

**Our Response:** We have strictly followed the PLOS ONE's style requirements, including:

1. For file naming

2. For code sharing

3. Included separate caption for each figure in the manuscript

4. Followed rules for specific citation if recommend by the reviewers

We hope our MS will get consideration for publication in the PLOS ONE

Yours Sincerely and Respectfully

Prof. Mohammad Nurul Matin

Department of Genetic Engineering and Biotechnology

University of Rajshahi, Rajshahi-6205, Bangladesh

**Reviewer's Responses to Editorial Questions and our Responses**

1. Is the manuscript technically sound, and do the data support the conclusions?

The manuscript must describe a technically sound piece of scientific research with data that supports the conclusions. Experiments must have been conducted rigorously, with appropriate controls, replication, and sample sizes. The conclusions must be drawn appropriately based on the data presented.

Reviewer #1: No

Reviewer #2: Yes

Reviewer #3: Partly

Reviewer #4: Yes

Our Response: Thank you for the observation. After careful revision and substantial improvement of the MS following comments and suggestions from reviewers, we hope the MS is better than the previous one for all aspects, including technical soundness and data consistency with the conclusions.

2. Has the statistical analysis been performed appropriately and rigorously?

Reviewer #1: N/A

Reviewer #2: I Don't Know

Reviewer #3: Yes

Reviewer #4: N/A

Our Response: Thank you for the observation. After substantial improvement following suggestions, now all aspects are appropriate and well described.

3. Have the authors made all data underlying the findings in their manuscript fully available?

The [PLOS Data policy](http://track.editorialmanager.com/CL0/http:%2F%2Fwww.plosone.org%2Fstatic%2Fpolicies.action%23sharing/1/010f01990ddef1e3-15882ca2-c8c0-452f-958a-6387a569aba0-000000/Vi2nKFCD4SZ9h6Hi8fFi4aLFvE0qhHme4Kk5pGfMvTI=227) requires authors to make all data underlying the findings described in their manuscript fully available without restriction, with rare exception (please refer to the Data Availability Statement in the manuscript PDF file). The data should be provided as part of the manuscript or its supporting information, or deposited to a public repository. For example, in addition to summary statistics, the data points behind means, medians and variance measures should be available. If there are restrictions on publicly sharing data—e.g. participant privacy or use of data from a third party—those must be specified.

Reviewer #1: No

Reviewer #2: Yes

Reviewer #3: Yes

Reviewer #4: No

Our Response: All data underlying the findings described in the manuscript are fully available without restriction. We have provided all data as part of the manuscript and its supporting information.

4. Is the manuscript presented in an intelligible fashion and written in standard English?

PLOS ONE does not copyedit accepted manuscripts, so the language in submitted articles must be clear, correct, and unambiguous. Any typographical or grammatical errors should be corrected at revision, so please note any specific errors here.

Reviewer #1: Yes

Reviewer #2: Yes

Reviewer #3: Yes

Reviewer #4: Yes

Our Response: Thank you for the assessment.

**5. Review Comments to the Author**
Please use the space provided to explain your answers to the questions above. You may also include additional comments for the author, including concerns about dual publication, research ethics, or publication ethics. (Please upload your review as an attachment if it exceeds 20,000 characters)

**Point-by-Point Response to the Comments from Reviewer #1**

**Q1**• The manuscript lacks a proper validation procedure for the docking studies. Redocking alone does not constitute sufficient validation. Structural superimposition of docked and co-crystallized ligands is recommended.

**Response:** Thank you for this important suggestion. In the revised manuscript (in the Results section, “Molecular Docking”), we have added a structural superimposition of the co-crystallized ligand with the re-docked pose. The RMSD between experimental and docked conformations was <2.0 Å, which demonstrates potentially increased reliability of the docking protocol. We hope this addition might provide stronger validation beyond redocking alone.

**Q2•** While the study refers to the use of deep learning, the number of ligands processed seems relatively limited or described method is not sufficient. A more comprehensive explanation or justification for labeling this approach as “deep learning” would be beneficial.

**Response:** We appreciate this observation and professional suggestion. We clarified in the revised manuscript (in the Methods section, “Deep Learning Screening”) that the model was trained on the CHEMBL4142 dataset comprising 891 known FGFR2 inhibitors, with ~880 molecular descriptors generated by PaDEL. The architecture (3 hidden layers, 2000–700–200 neurons) and activation functions follow standard deep learning frameworks. Although only the top 16 compounds were re-screened, the term “deep learning” is justified by the multilayer neural network model applied to a large training dataset. This clarification has been added to avoid misunderstanding. The DeepScreening platform provides an integrated framework for deep learning–based virtual screening. Its workflow involves: (i) dataset preparation, where a specific target is selected to train the deep neural network (DNN); (ii) feature selection, in which molecular descriptors are identified and vectorized; (iii) model parameterization, where essential parameters are defined for training regression models; and (iv) virtual screening, in which the trained model is applied to evaluate large chemical libraries. DeepScreening is a fully automated and advanced server that combines data preprocessing, model construction, and virtual screening into a seamless pipeline.

**Q3•** The authors mention the use of SwissADME, pkCSM, and ProTox-III for ADMET. However, the results from these tools are not fully presented, nor is there a comparative analysis. If a detailed comparison is not intended than result from single server may suffice.

**Response:** Thank you for this valuable point. In the revised manuscript, we have clarified the ADME/T analysis by specifying that SwissADME was used for pharmacokinetic and physicochemical property predictions, while ProTox-III was employed for toxicity assessment. We hope this additional description of the results comparisons may represent the complete content.

**Q4•** The software used to generate Figure 2 has not been identified.

**Response:** Thank you for noticing this unintentional omission. We have specified in the revised figure legend that chemical structures were generated using the 2D Sketcher (Beta) software. (Figure 2).

**Q5•** In the supplementary table, it would be helpful to include the proper botanical names, italics, authority.

**Response:** We agree with this suggestion. The supplementary table (S1 Table) has been revised to include the full botanical names with authorities in italics, following standard nomenclature guidelines.

**Q6•** Many known or previously reported compounds appear to be missing from the listed plant species. It is recommended to cross-reference the identified compounds with databases such as PubChem or relevant literature sources, and to include PubChem IDs or citations where applicable.

**Response:** We thank the reviewer for this suggestion. In the revised S1 Table, we cross-checked compounds against PubChem and relevant phytochemical databases, and we added PubChem CIDs for each compound. Missing compounds that were previously overlooked have also been included where relevant.

**Some typos like**
**•** In line 29, the phrase “a library of 1,350 phytochemicals derived from 51 anti-cancer medicinal plants” could be reconsidered as “plants with reported anticancer properties or plants traditionally used for anticancer purposes.”

**Response:** Thank you for this wording suggestion. We revised the sentence in the Abstract and methods accordingly to “plants with reported anticancer properties or traditionally used for anticancer purposes,” which is more precise.

**•** Although emphasis appears to have been placed on certain compounds by using capital letters, compound names such as uralenol, glycyrol, and abyssinone should conventionally be written in lowercase.

**Response:** We appreciate this correction. All compound names in the manuscript have been standardized to lowercase in accordance with chemical naming conventions.

We hope our MS will get consideration for publication in the PLOS ONE

Yours Sincerely and Respectfully

Prof. Mohammad Nurul Matin

Department of Genetic Engineering and Biotechnology

University of Rajshahi, Rajshahi-6205, Bangladesh

**Point-by-Point Response to the Comments from Reviewer #2**

**Comment:** Fibroblast Growth Factor Receptor 2 (FGFR2), which is essential for cellular proliferation and differentiation and whose dysregulation is linked to a number of malignancies, is the subject of your research. Using a library of 1,350 phytochemicals extracted from 51 anti-cancer medicinal plants, your study combines molecular docking, deep learning, pharmacokinetic profiling, and molecular dynamics (MD) simulations to find putative FGFR2 inhibitors. These elements may be used therapeutically to treat cancer.

**Response:** We thank the reviewer for their encouraging assessment. We are pleased that the study design combining docking, deep learning, ADME/T, and MD simulation was considered scientifically sound. We have revised the manuscript for greater clarity and completeness in response to all reviewer suggestions.

We hope our MS will get consideration for publication in the PLOS ONE

Yours Sincerely and Respectfully

Prof. Mohammad Nurul Matin

Department of Genetic Engineering and Biotechnology

University of Rajshahi, Rajshahi-6205, Bangladesh

**Point-by-Point Response to the Comments from Reviewer #3**

**Comment:** This manuscript presents a thorough and comprehensive study focused on identifying potential FGFR2 inhibitors using advanced computational methods including molecular docking, deep learning, pharmacokinetic profiling, and molecular dynamics simulations. The manuscript is well-structured and logical, and the conclusions are well-supported by the presented data. The authors have appropriately framed the analytical approach and have acknowledged the study’s limitations, such as the need for experimental validation.
However, it is emphasized that the findings are computational predictions, and further experimental in vitro and in vivo validation is essential for confirming these results. It is also recommended that more detailed information about the parameters used in the simulations and deep learning models be included in the methodology section to ensure reproducibility.
No ethical concerns regarding publication or originality were noted, and all data have been provided fully and transparently. The manuscript is clearly written in scientific language and requires no major language corrections.

Overall, this manuscript is a valuable contribution toward targeted drug development for FGFR2, and I recommend its acceptance pending minor revisions to include additional methodological details and a clear statement on the necessity of experimental validation.

**Response:** We sincerely thank the reviewer for the thoughtful and encouraging evaluation of our manuscript. We appreciate the recognition of the study’s comprehensive approach and the constructive suggestions provided. As our findings are based on computational predictions and we have added a clear statement accordingly emphasizing the necessity of future in vitro and in vivo validation studies to confirm our results, however, we could not perform a lab experiment at this time due to a lack of research funding. We hope that future work will focus on experimental validation once resources are available.

We agree with the reviewer’s recommendation to include more detailed information on the parameters used in the molecular docking, molecular dynamics simulations, and deep learning models. We have revised the Methods section (Deep Learning and MD Simulation) to provide explicit descriptions of these parameters to ensure clarity and reproducibility.

We hope our MS will get consideration for publication in the PLOS ONE

Yours Sincerely and Respectfully

Prof. Mohammad Nurul Matin

Department of Genetic Engineering and Biotechnology

University of Rajshahi, Rajshahi-6205, Bangladesh

**Point-by-Point Response to the Comments from Reviewer #4**

**comments:**
**1.** The methods section states, "A comprehensive literature review was conducted to collect a list of 51 medicinal plants with anticancer properties, along with their 1,350 phytochemicals (S1 Table) identified based on GC/MS analysis and downloaded in SDF format from the PubChem database." Kindly explain the parameters used to determine the criteria for selecting phytochemicals.

**Response:** We sincerely thank the reviewer for this question and suggestion. Selection was based on the reported anticancer activity of plants in published literature, followed by the collection of all identified phytochemicals via GC/MS studies. We have further clarified these parameters for selecting phytochemicals in the Method section.

**2.** The method states, "The ADMET properties 158 were forecasted using SwissADME, pKCSM, and ProTox-III servers." Kindly explain what parameters were analyzed from each web server used.

**Response:** We revised the Methods to clarify: SwissADME was used for pharmacokinetic and physicochemical properties (MW, LogP, TPSA, solubility, Lipinski rules, absorption, distribution, metabolism, excretion); and ProTox-III for toxicity predictions (LD50, mutagenicity, hepatotoxicity, carcinogenicity).

**3.** In the results, it is stated "RMSD, RMSF, radius of gyration (Rg), solvent-accessible surface area (SASA), free energy landscape, and principal component analysis (PCA)". In addition to that, kindly also includes simulations for MM-PBSA/GBSA for more accurate molecular dynamics results compared to docking scores.

**Response:** We appreciate and thank the reviewer for this valuable suggestion regarding the inclusion of MM-PBSA/GBSA for more accuracy of the results. We fully agree that MM-PBSA/GBSA would provide deeper insight into binding affinities beyond docking scores. However, due to current computational and funding limitations, we were unable to perform MM-PBSA/GBSA calculations at this stage. We have acknowledged this as a limitation in the Discussion section and suggested it as an important next step for future work to strengthen binding energy validation.

**4.** In the discussion, Table 3 should be moved to the results.

**Response:** We have moved the Table 3 (toxicity analysis) to the Results section, as recommended.

We hope our MS will get consideration for publication in the PLOS ONE

Yours Sincerely and Respectfully

Prof. Mohammad Nurul Matin

Department of Genetic Engineering and Biotechnology

University of Rajshahi, Rajshahi-6205, Bangladesh

6. PLOS authors have the option to publish the peer review history of their article ([what does this mean?](https://track.editorialmanager.com/CL0/https:%2F%2Fjournals.plos.org%2Fplosone%2Fs%2Feditorial-and-peer-review-process%23loc-peer-review-history/1/010f01990ddef1e3-15882ca2-c8c0-452f-958a-6387a569aba0-000000/geILmuaQUs5NiWdQ7V2nQpVSjnwRmrbVnbBeDhElJtY=227)). If published, this will include your full peer review and any attached files.

If you choose “no”, your identity will remain anonymous but your review may still be made public.

**Do you want your identity to be public for this peer review?** For information about this choice, including consent withdrawal, please see our [Privacy Policy](https://track.editorialmanager.com/CL0/https:%2F%2Fwww.plos.org%2Fprivacy-policy/1/010f01990ddef1e3-15882ca2-c8c0-452f-958a-6387a569aba0-000000/jiwpKUhc3CWY4xLzPjGkzLwpJtvFkGCSsTJgTU_fMSI=227).

Reviewer #1: No

Reviewer #2: **Yes:** Dr. Vijay Vardhan Pandey

Reviewer #3: **Yes:** Mehdi Ghasemi Nafchi

Reviewer #4: No

While revising your submission, please upload your figure files to the Preflight Analysis and Conversion Engine (PACE) digital diagnostic tool, <https://pacev2.apexcovantage.com/>. PACE helps ensure that figures meet PLOS requirements. To use PACE, you must first register as a user. Registration is free. Then, login and navigate to the UPLOAD tab, where you will find detailed instructions on how to use the tool. If you encounter any issues or have any questions when using PACE, please email PLOS at [figures@plos.org](mailto:figures@plos.org). Please note that Supporting Information files do not need this step.

**Answer to the Additional comments from R#3**

**Q1.** **Title and Abstract**

The title is clear, concise, and appropriately reflects the topic and objective of the research (page 1). The abstract is comprehensive, including the aim, methods, key results, and conclusions; it effectively summarizes the main points (page 2). The term "promising" in the abstract could be more cautiously worded, specifying the preliminary nature of the findings.

**Response:** We agree with the reviewer’s observation. In the revised Abstract, we have replaced “promising inhibitors” with “potential inhibitors requiring further experimental validation.” This revision better reflects the preliminary computational nature of our findings.

**Q2. Introduction**

The scientific rationale is well presented, highlighting the importance of FGFR2 in several cancers (pages 2 to 4). A thorough review of prior studies and the role of FGFR2 is provided. Some parts of the introduction are somewhat lengthy and repetitive; for example, detailed signaling pathway descriptions could be more concise.

**Response:** Thank you for this suggestion. We carefully revised the Introduction to remove redundancies and condensed the detailed descriptions of signaling pathways. This has improved the readability and conciseness of the section while preserving key scientific information.

**Q3. Materials and Methods**

Detailed and clear descriptions of the 3D structure preparation of FGFR2 and ligand preparations are provided (pages 5 to 7). Software and parameters used are well-documented. The docking process using AutoDock Vina is appropriately executed. Selection of compounds based on binding energy and detailed interaction descriptions are thorough. More explicit comparison of docking results with reference drugs would strengthen validation.

Use of RNN and CHEMBL4142 dataset for predicting pIC50 is well described. Model performance metrics (R²=0.68, MSE, RMSE) are satisfactorily reported (page 11). Further discussion on the model limitations and their impact on results is recommended.

Utilization of three reputable servers to predict ADMET properties is provided, with clear explanation of physicochemical properties, absorption, and toxicity in Tables 2 and 3 (pages 16 to 18). Some ADME/T specialized terms could be briefly explained for a broader readership.

In Molecular Dynamics Simulation (MD), a 100 ns simulation with thorough details on parameters and methodology is performed. System preparation and computational parameters are comprehensively documented (pages 7 to 10). Adding more comparative graphs within the text might assist reader understanding.

**Response:** We appreciate these constructive suggestions. In the Methods and Results sections of the revised manuscript, we have made most corrections as follows:

- We had added in the revised version of the manuscript a comparison of docking scores of the selected phytochemicals with known FGFR2 inhibitors (Fgfr3-IN-2) as references, strengthening the validation of our results (See Table 1).
- Limitations of the deep learning model (e.g., reliance on available datasets, potential bias in chemical space) are now discussed in the Methods and Discussion sections.
- Explanation of ADME/T term has been discussed, as well as others like TPSA, GI absorption, and BBB permeability have been added in footnotes to Tables 2 and 3 for clarity.
- In the revised manuscript, we clearly describe on MD graphs of all parts separately in the Results to better understanding for general reader.

**Q4. Results**

Clear ranking of top compounds with binding energies and molecular interaction visuals (Tables 1, Figures 1 and 2). Presentation of deep learning results, ADME/T analysis, and MD simulation data with RMSD, RMSF, Rg, SASA, and PCA plots (Figures 3 to 5). Well-interpretated results. More extensive comparison with similar studies would enhance the novelty and significance context.

**Response:** Thank you for this valuable point. In the Discussion section of the revised version, we have added some references to recent computational studies on FGFR inhibitors and compared our outcomes to highlight similarities and differences. This addition contextualizes our results and emphasizes the novelty of identifying uralenol, glycyrol, and abyssinone II as potential FGFR2 inhibitors.

**Q5. Discussion**

The importance of FGFR2 is adequately discussed, including up-to-date therapeutic perspectives (pages 21 to 27). Limitations are transparently acknowledged, particularly on absence of experimental validation and reliance on computational predictions. More concrete discussion on the potential clinical applications and recommended future experimental directions are encouraged.

**Response:** We appreciate this comment. In the revised Discussion, we expanded on the translational potential of FGFR2 inhibitors in precision oncology. We also outlined specific experimental directions, such as in vitro kinase inhibition assays, cell viability tests in FGFR2-overexpressing cancer cell lines, and in vivo xenograft models, as essential next steps to validate our computational predictions.

**Q6. Conclusion**

Conclusions are clear, justified by key findings. Emphasis on the need for experimental validation for clinical progression is appropriate (pages 27 to 29).

**Response:** We thank the reviewer for the positive assessment of the Conclusion section.

**Q7. Writing Suggestions:**

Long sentences in “Introduction” could be divided into shorter ones to improve readability.

**Response:** We appreciate the reviewer’s suggestion. In the revised manuscript, we have carefully edited the “Introduction” section by breaking down long sentences into shorter ones to enhance clarity and readability.

**Q8. Ethical Considerations and Data Availability**

Explicit clarification of ethical approval status (especially if no human/animal data used) would improve transparency.

**Response:** We confirm that this study did not involve human participants, animal experiments, or clinical data; therefore, ethical approval was not required. We have clarified this point in the “Ethical Statement” section of the revised manuscript to ensure transparency.

**Overall,** this article presents a scientifically sound, well-documented, and advanced computational study of FGFR2 inhibitors. With minor editorial revisions, it is suitable for publication in PLOS ONE and will contribute valuable insight to the field.

**Response:** We sincerely thank the reviewer for their positive and encouraging evaluation of our manuscript. We are pleased that our work is considered scientifically sound and valuable. All reviewers' and editorial issues have been carefully revised as suggested to improve clarity, consistency, and readability. We greatly appreciate the reviewer’s constructive feedback and support for the publication of our work in PLOS ONE.

We hope our MS will get consideration for publication in the PLOS ONE

Yours Sincerely and Respectfully

Prof. Mohammad Nurul Matin

Department of Genetic Engineering and Biotechnology

University of Rajshahi, Rajshahi-6205, Bangladesh
