# Supplementary material for: Uralenol, Glycyrol, and Abyssinone II as potent inhibitors of fibroblast growth factor receptor 2 from anti-cancer plants: A deep learning and molecular dynamics approach
Source: PLoS One. 2026 Jan 30;21(1):e0341498. doi: 10.1371/journal.pone.0341498 (PMC12857956; doi:10.1371/journal.pone.0341498)
Supplement: Attachment — Submitted filename: Response_to_Reviewers_auresp_2.docx [file pone.0341498.s004.docx]

**Cover Letter	December 27, 2025**

**Ref.:** PONE-D-25-37887R1

**Title:** Uralenol, Glycyrol, and Abyssinone II as potent inhibitors of Fibroblast Growth Factor Receptor 2 from anti-cancer plants: a deep learning and molecular dynamics approach

**Journal:** Plos One

Dear editor and reviewer,

Thank you for further reviewing our manuscript (MS). We are sincerely grateful for your careful and constructive comments, as well as your valuable suggestions, which have greatly strengthened the MS. According to your suggestions and recommendations, we have thoroughly revised the MS. All amendments were made point by point in response to your suggestions, and the revised sections have been highlighted in RED.

Thank you and best regards.

Yours Sincerely and Respectfully

Prof. Mohammad Nurul Matin

Department of Genetic Engineering and Biotechnology

University of Rajshahi, Rajshahi-6205, Bangladesh

**Point-by-Point Response to Editorial Comments**

**Editorial comments**

Dear Dr. Matin,

Thank you for submitting your manuscript to PLOS ONE. After careful consideration, we feel that it has merit but does not fully meet PLOS ONE’s publication criteria as it currently stands. Therefore, we invite you to submit a revised version of the manuscript that addresses the points raised during the review process.

Please submit your revised manuscript by Feb 08 2026 11:59PM. If you will need more time than this to complete your revisions, please reply to this message or contact the journal office at [plosone@plos.org](mailto:plosone@plos.org). When you're ready to submit your revision, log on to <https://www.editorialmanager.com/pone/> and select the 'Submissions Needing Revision' folder to locate your manuscript file.

Please include the following items when submitting your revised manuscript:

- A letter that responds to each point raised by the academic editor and reviewer(s). You should upload this letter as a separate file labeled 'Response to Reviewers'.
- A marked-up copy of your manuscript that highlights changes made to the original version. You should upload this as a separate file labeled 'Revised Manuscript with Track Changes'.
- An unmarked version of your revised paper without tracked changes. You should upload this as a separate file labeled 'Manuscript'.

We look forward to receiving your revised manuscript.

Kind regards,

Jorddy Neves Cruz
Academic Editor, PLOS ONE

**Our Response:** We would like to express our gratitude for the consideration of our work with the decision “Minor Revision”. Significant revisions with corresponding corrections addressing comments and suggestions from the editor and reviewers have been incorporated into the revised MS. We have provided a point-by-point response to each question from the referees. All modifications to the MS are highlighted in red, so that any changes can be easily reviewed.

We have carefully followed the journal guidelines in the revised version and submitted the MS within the mentioned deadline. While submitting, we have uploaded all the necessary item files labeled with:

- 'Response to Reviewers’
- 'Revised Manuscript with Track Changes’
- Unmarked manuscript labeled ‘Revised Manuscript-clean’

Also, we have followed the guidelines for resubmitting figure files.

**Journal Requirements:**

1. If the reviewer comments include a recommendation to cite specific previously published works, please review and evaluate these publications to determine whether they are relevant and should be cited. There is no requirement to cite these works unless the editor has indicated otherwise.

2. Please review your reference list to ensure that it is complete and correct. If you have cited papers that have been retracted, please include the rationale for doing so in the manuscript text, or remove these references and replace them with relevant current references. Any changes to the reference list should be mentioned in the rebuttal letter that accompanies your revised manuscript. If you need to cite a retracted article, indicate the article’s retracted status in the References list and also include a citation and full reference for the retraction notice.

**Our Response:** We have strictly followed the PLOS ONE's requirements, including:

1. Followed rules for specific citation if recommended by the reviewers
2. We reviewed the reference list to ensure that it is complete and correct. After careful checking, we found that no one has been retracted from the web.

We hope our MS will get consideration for publication in the PLOS ONE

Yours Sincerely and Respectfully

Prof. Mohammad Nurul Matin

Department of Genetic Engineering and Biotechnology

University of Rajshahi, Rajshahi-6205, Bangladesh

**Reviewer's Responses to Editorial Questions and our Responses**

1. If the authors have adequately addressed your comments raised in a previous round of review and you feel that this manuscript is now acceptable for publication, you may indicate that here to bypass the “Comments to the Author” section, enter your conflict of interest statement in the “Confidential to Editor” section, and submit your "Accept" recommendation.?

Reviewer #4: All comments have been addressed

Reviewer #5: All comments have been addressed

Our Response: We sincerely thank the reviewers for their careful evaluation of our article and responses to those we have submitted in the previous submission.

2. Is the manuscript technically sound, and do the data support the conclusions?

Reviewer #4: Yes

Reviewer #5: Yes

Our Response: We sincerely thank the reviewers for their careful evaluation.

3. Has the statistical analysis been performed appropriately and rigorously?

Reviewer #4: N/A

Reviewer #5: N/A

4. Have the authors made all data underlying the findings in their manuscript fully available?

Reviewer #4: No

Reviewer #5: Yes

Our Response: All data underlying the findings described in the manuscript are fully available without restriction. We have provided all data as part of the manuscript and its supporting information.

5. Is the manuscript presented in an intelligible fashion and written in standard English?

Reviewer #4: Yes

Reviewer #5: Yes

Our Response: Thank you for the assessment.

**6. Review Comments to the Author**

**Point-by-Point Response to the Comments from Reviewer #4**

**comments:** The authors have addressed all previous comments with satisfactory answers and corrections. I can recommend the editors to accept this article.

**Our Response:** We sincerely thank the reviewer for your positive evaluation and constructive feedback. We are pleased that the revisions and clarifications have satisfactorily addressed all previous comments. We greatly appreciate the reviewer’s recommendation for acceptance.

**Point-by-Point Response to the Comments from Reviewer #5**

**Reviewer Comments: Minor Revisions**
The combination of molecular docking, deep learning, ADME/T profiling, and molecular dynamics simulations creates a comprehensive in-silico framework for discovering possible FGFR2 inhibitors. The study is interesting and provides useful insights; nevertheless, a few small changes are required to improve clarity and strengthen the paper.
Our Response: We sincerely thank the reviewer for the positive evaluation of our work and for recognizing the comprehensive in-silico framework employed in this study. We appreciate the constructive suggestions provided to improve clarity and strengthen the manuscript. All minor comments have been carefully addressed, and the manuscript has been revised accordingly to enhance readability and scientific rigor.

**Q1. Clarify the abbreviations in the abstract.**
Abbreviations, such as pIC₅₀ and PCA, should be defined at the beginning of the abstract. This will improve accessibility for readers who are unfamiliar with these terminology.

Our Response: We thank the reviewer for this valuable suggestion. All abbreviations used in the abstract, including pIC₅₀ (negative logarithm of the half-maximal inhibitory concentration) and PCA (principal component analysis), as well as in the text, have now been clearly defined at their first appearance to improve clarity and accessibility for readers.

**Q2. Strengthen the discussion section.**
A more in-depth contextualization might improve the conversation. Please compare your findings to previous studies on FGFR2 inhibitors and phytochemical-based kinase inhibitors.
Highlight how your identified compounds (uralenol, glycyrol, and abyssinone II) compare to or differ from previously reported inhibitors in terms of binding affinity, stability, and projected pharmacokinetics.

Our Response: We sincerely thank the reviewer for the professional suggestion to strengthen the discussion by providing deeper contextualization of our findings relative to previous work. In the revised manuscript, we have expanded the Discussion, including comparative analysis of uralenol, glycyrol, and abyssinone II with previously reported FGFR2 inhibitors and phytochemical-based kinase inhibitors. Specifically, we now discuss how the binding affinities and interaction profiles of these compounds relate to those reported in the literature. These additions provide a clearer context for our results and more thoroughly integrate our findings with existing research.

**Changes made:** The following paragraph with proper citation has been added to the Discussion section:

Recent studies on FGFR2 inhibitors report binding affinities of -8.0 to -9.6 kcal/mol for synthetic compounds (<https://doi.org/10.1016/j.imu.2023.101368>, and −8.3801 kcal/mol, −8.3014 kcal/mol, and −8.320 kcal/mol for Knipholone, Torosanin, and Rubrolide E, respectively <https://doi.org/10.1016/j.ijbiomac.2025.146888>). Our phytochemicals—uralenol (-9.8 kcal/mol), glycyrol (-9.7 kcal/mol), and abyssinone II (-9.7 kcal/mol)—show better competitive binding. Clinical FGFR inhibitors like erdafitinib and infigratinib exhibit moderate structural stability in MD simulations (<https://doi.org/10.1007/s10549-015-3301-y>, <https://doi.org/10.1038/s41416-020-01157-0>), whereas our compounds demonstrated superior stability (RMSD: 1.21-1.88 Å). Previous phytochemical studies targeting FGFRs showed weaker activity (<https://doi.org/10.3390/cancers12103029>). Unlike synthetic inhibitors facing selectivity and resistance challenges (<https://doi.org/10.7150/ijbs.20792>), our compounds exhibited favorable ADME properties with low toxicity predictions. These natural compounds present promising alternatives to current FGFR2 therapeutics, combining strong binding affinity, enhanced stability, and superior safety profiles.

**Q3. Include a limitation section.**
The manuscript currently lacks a clear limitation statement. Please include a section to acknowledge that: The investigation is completely in silico, No experimental validation (in vitro or in vivo) was conducted, and As a result, the findings should be regarded predictively rather than confirmatory.

Our Response: We would like to express our sincere thanks to the reviewer for this important suggestion. We agree with this inoculation. A dedicated l**imitation statement** has now been added in the text, acknowledging that the present investigation is entirely in silico, that no experimental validation was performed, and that the results should therefore be interpreted as predictive rather than confirmatory. We also emphasize that experimental studies will be required in future work to validate the computational findings.

**Addition made:**

(This study is entirely computational and lacks experimental validation. While molecular docking, deep learning, and molecular dynamics simulations provide valuable predictive insights, *in vitro* and *in vivo* studies are essential to confirm biological efficacy and therapeutic potential. The ADME/T and toxicity profiles were predicted using *in silico* models, which may not fully reflect complex human physiological responses. The focus on a subset of top-ranked compounds may have excluded other potential candidates. Therefore, our findings should be regarded as predictive rather than confirmatory. Expanding the chemical space and conducting experimental validation, such as *in vitro* kinase inhibition assays, cell viability tests in FGFR2-overexpressing cancer cell lines, and *in vivo* xenograft models, are essential next steps to validate these computational predictions and advance these compounds toward preclinical development).

**Q4. Improve the conclusion.**
The conclusion should be concise and properly articulated: The major findings, the novelty of the identified compounds, and the next steps required for experimental validation.

Our Response: We thank the reviewer for this helpful suggestion. The Conclusion section has been revised to be more concise and clearly articulated. It now summarizes the major findings of the study, highlights the novelty of the identified compounds as potential FGFR2 inhibitors, and outlines the necessary next steps for experimental validation, including in vitro and in vivo studies. This revision improves clarity and emphasizes the translational relevance of the work.

**Changes made:** The Conclusion has been revised as follows:

This study successfully identified uralenol, glycyrol, and abyssinone II as potent and stable FGFR2 inhibitors from a phytochemical library of traditionally used anti-cancer plants using an integrated in silico framework. The integrated approach of molecular docking, deep learning prediction, ADME/T evaluation, and molecular dynamics simulations provided strong evidence for the structural stability, binding affinity, bioactivity, and favorable pharmacokinetic properties of these compounds toward FGFR2. Competitive binding affinities (-9.7 to -9.8 kcal/mol) comparable to synthetic inhibitors, superior structural stability (RMSD: 1.21-1.88 Å) during MD simulations, favorable drug-like properties with minimal predicted toxicity, and stable conformational dynamics confirmed by PCA and free energy landscape analyses further confirmed stable and energetically favorable protein–ligand interactions. Importantly, this work highlights the novel potential of these phytochemicals as FGFR2-targeted anticancer candidates, expanding the scope of plant-derived kinase inhibitors with enhanced stability and safety profiles compared to current synthetic therapeutics, addressing limitations of off-target toxicity and resistance. These computational predictive findings support the therapeutic potential of the compounds and highlight them as potential candidates for further validation through *in vitro* kinase inhibition assays against FGFR2, cell viability and proliferation studies in FGFR2-overexpressing cancer cell lines, and *in vivo* xenograft models to assess antitumor efficacy. Successful experimental validation could position these natural compounds as safer alternatives for FGFR2-targeted cancer therapy.

We hope our MS will get consideration for publication in the PLOS ONE

Yours Sincerely and Respectfully

Prof. Mohammad Nurul Matin

Department of Genetic Engineering and Biotechnology

University of Rajshahi, Rajshahi-6205, Bangladesh
